# Changes in Resting and Exercise Hemodynamics Early After Heart Transplantation: A Simulation Perspective

**DOI:** 10.3389/fphys.2020.579449

**Published:** 2020-11-06

**Authors:** Max Haberbusch, Daniela De Luca, Francesco Moscato

**Affiliations:** ^1^Center for Medical Physics and Biomedical Engineering, Medical University of Vienna, Vienna, Austria; ^2^Ludwig Boltzmann Institute for Cardiovascular Research, Vienna, Austria; ^3^BioRobotics Institute, Scuola Superiore Sant’Anna, Pisa, Italy; ^4^Department of Information Engineering, University of Pisa, Pisa, Italy; ^5^Austrian Cluster for Tissue Regeneration, Vienna, Austria

**Keywords:** heart transplantation, cardiac denervation, hemodynamics, exercise response, numerical model, computer simulation

## Abstract

**Introduction:** During heart transplantation (HTx), cardiac denervation is inevitable, thus typically resulting in chronic resting tachycardia and chronotropic incompetence with possible consequences in patient quality of life and clinical outcomes. To this date, knowledge of hemodynamic changes early after HTx is still incomplete. This study aims at providing a model-based description of the complex hemodynamic changes at rest and during exercise in HTx recipients (HTxRs).

**Materials and Methods:** A numerical model of early HTxRs is developed that integrates intrinsic and autonomic heart rate (HR) control into a lumped-parameter cardiovascular system model. Intrinsic HR control is realized by a single-cell sinoatrial (SA) node model. Autonomic HR control is governed by aortic baroreflex and pulmonary stretch reflex and modulates SA node activity through neurotransmitter release. The model is tuned based on published clinical data of 15 studies. Simulations of rest and exercise are performed to study hemodynamic changes associated with HTxRs.

**Results:** Simulations of HTxRs at rest predict a substantially increased HR [93.8 vs. 69.5 beats/min (bpm)] due to vagal denervation while maintaining normal cardiac output (CO) (5.2 vs. 5.6 L/min) through a reduction in stroke volume (SV) (55.4 vs. 82 mL). Simulations of exercise predict markedly reduced peak CO (13 vs. 19.8 L/min) primarily resulting from diminished peak HRs (133.9 vs. 169 bpm) and reduced ventricular contractility. Yet, the model results show that HTxRs can maintain normal CO for low- to medium-intensity exercise by increased SV augmentation through the Frank–Starling mechanism.

**Conclusion:** Relevant hemodynamic changes occur after HTx. Simulations suggest that (1) increased resting HRs solely result from the absence of vagal tone; (2) chronotropic incompetence is the main limiting factor of exercise capacity whereby peripheral factors play a secondary role; and (3) despite the diminished exercise capacity, HTxRs can compensate chronotropic incompetence by a preload-mediated increase in SV augmentation and thus maintain normal CO in low- to medium-intensity exercise.

## Introduction

Heart transplantation (HTx) is the last resort for an increasing number of persons suffering from end-stage heart failure. During HTx, sympathetic and vagal denervation of the heart is inevitable, leading to postoperative chronotropic incompetence and thus to reduced quality of life (Awad et al., 2016). Especially in the first year after HTx, patients suffer from chronic tachycardia, with resting heart rates (HRs) elevated to greater than 90 beats/min (bpm) and significantly reduced HR variability (HRV) (Awad et al., 2016; Kobashigawa and Olymbios, 2017). Furthermore, on top of raised HR recovery times, HTx recipients (HTxRs) show delayed and impeded exercise response (Awad et al., 2016; Kobashigawa and Olymbios, 2017), reaching peak HRs as low as only 133 bpm (McLaughlin et al., 1978; Crisafulli et al., 1985; Kavanagh et al., 1988; Labovitz et al., 1989; Wilson et al., 1991; Marzo et al., 1992; Rudas et al., 1993; Kao et al., 1994; Doering et al., 1996; Geny et al., 1996; Notarius et al., 1998; Hayman et al., 2010; Peled et al., 2017; Nygaard et al., 2019; Nytrøen et al., 2019). According to literature, elevated resting HRs are most certainly a result of the absence of vagal tone, rendering the heart to rely on intrinsic control only, whereas delayed and impeded exercise response is most likely due to lack of vagal withdrawal and missing sympathetic drive (Awad et al., 2016). The exercise response of HTxR is thought to depend solely upon circulating catecholamines and a strong reliance on the Frank–Starling mechanism to compensate for the impaired autonomic response (Awad et al., 2016). Ultimately, elevated resting HRs to greater than 90 bpm and increased postexercise HR recovery times were shown to be strongly correlated with raised mortality (Awad et al., 2016; Kobashigawa and Olymbios, 2017).

With globally increasing prevalence, cardiovascular diseases are still the leading cause of mortality, projected to be responsible for more than 25 million global deaths by 2030 (Okwuosa et al., 2016; Timmis et al., 2020). This situation combined with increased donor organ availability leads to a steady increase of currently 5,500 annual HTx worldwide (Khush et al., 2019). Consequently, there is a major need for treatment modalities that help to improve the quality of life and clinical outcomes early after HTx (Awad et al., 2016). However, to this date, still, there is a substantial lack of knowledge on the mechanisms associated with cardiac denervation and spontaneous reinnervation in HTxRs (Awad et al., 2016), thus restraining the development of new therapies to provide relief for those affected. Therefore, tools and frameworks that facilitate the investigation of physiological changes in HTxRs to find potential treatment modalities are of constant need and great significance.

In recent years, numerical simulations have gained increasing popularity in cardiovascular research, especially to gain a better understanding of the human hemodynamics and its changes associated with various diseases (Jung et al., 2006; Banerjee et al., 2008; Qian et al., 2010; Politi et al., 2016). Moreover, mathematical modeling was successfully proven to be an effective technique for the design and evaluation of potential treatment modalities for heart failure patients, such as mechanical circulatory assist devices (Moscato et al., 2010, 2013; Gross et al., 2020).

Although there are various numerical attempts to study the cardiac autonomic neural regulation (Levy and Zieske, 1969; Katona et al., 1970; Ursino and Magosso, 2003; Kember et al., 2014, 2017), to the best of our knowledge, up until now, there is no literature concerned with the simulation of hemodynamic changes and alterations of autonomic cardiac control associated with HTxRs. Therefore, based on published clinical data, we developed an integrated numerical model that is capable of predicting resting and exercise hemodynamics of HTxRs with good accuracy. The model does not attempt to represent a single patient’s behavior but rather cohort hemodynamics.

## Materials and Methods

**FIGURE 1:**
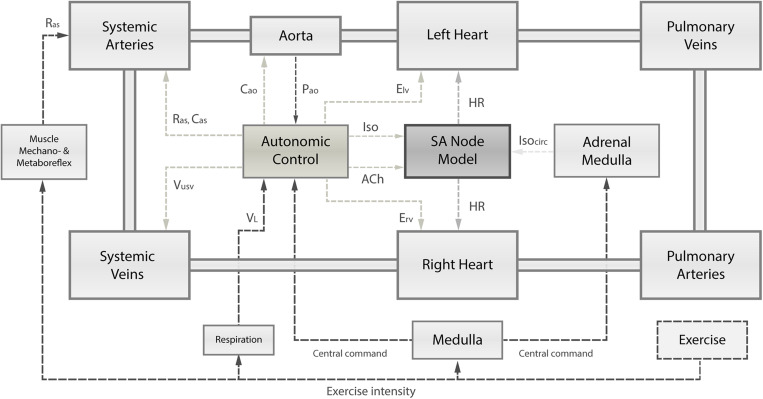
Schematic overview of the integrated numerical model, comprising (1) the cardiovascular system and its principal compartments; (2) the single-cell SA node model; and (3) the autonomic control governed by arterial baroreflex and pulmonary stretch reflex integrating receptor information on instantaneous aortic pressure (*P*_ao_) and instantaneous lung volume (*V*_L_). Based on autonomic activity, the SA node depolarization rate is modulated through changes in acetylcholine (ACh) and isoprenaline (Iso) concentrations. Autonomic control has also influence on the left and right ventricular contractility (*E*_lv_, *E*_rv_), systemic vascular resistance (*R*_as_), and systemic venous unstressed volume (*V*_usv_). Muscle mechanoreflex and metaboreflex modulate Ras to account for vasodilation in response to exercise. Exercise intensity determines the level of central command, respiration frequency, and muscle mechanoreflex and metaboreflex activity. Ultimately, the central command has a direct influence on autonomic control loops and the adrenal medulla releasing catecholamines (Iso_*circ*_).

We present an integrated numerical model that was modified to reproduce the changes in hemodynamic parameters observed in early HTxRs, both at rest and during exercise. The proposed model integrates three closely linked components: (1) a closed-loop lumped parameter model of the human cardiovascular system; (2) the intrinsic HR control represented by a Hodgkin-Huxley-type single-cell sinoatrial (SA) node model; and (3) the autonomic cardiac control mediated by arterial baroreflex and the pulmonary stretch reflex. A schematic overview of the model structure can be found in Figure 1. Finally, the model parameters were adjusted based on clinical data from 15 publications (McLaughlin et al., 1978; Crisafulli et al., 1985; Kavanagh et al., 1988; Labovitz et al., 1989; Wilson et al., 1991; Marzo et al., 1992; Rudas et al., 1993; Kao et al., 1994; Doering et al., 1996; Geny et al., 1996; Notarius et al., 1998; Hayman et al., 2010; Peled et al., 2017; Nygaard et al., 2019; Nytrøen et al., 2019) that were identified in a careful literature review. The model equations were implemented in MathWorks^®^ SIMULINK^®^ 9.1 (2019a) and numerically integrated using the ode15s solver with a maximum step size of 0.01 s, and an absolute error tolerance of 10^–3^. All parameter values used to simulate HTxRs and an age- and gender-matched healthy control group may be found in Supplementary Tables S2–S8.

### Model Architecture

#### Cardiovascular System

To simulate the human cardiovascular system, the numerical closed-loop lumped-parameter model published by Moscato et al. (2010, 2013) was modified to reproduce the hemodynamics of early HTxRs and demographically matched healthy cohorts reported in the literature (McLaughlin et al., 1978; Crisafulli et al., 1985; Kavanagh et al., 1988; Labovitz et al., 1989; Wilson et al., 1991; Marzo et al., 1992; Rudas et al., 1993; Kao et al., 1994; Doering et al., 1996; Geny et al., 1996; Notarius et al., 1998; Hayman et al., 2010; Peled et al., 2017; Nygaard et al., 2019; Nytrøen et al., 2019). The hemodynamic model comprises atria and ventricles represented by time-varying non-linear elastance models, heart valves realized by diode resistance series, and compartments for systemic and pulmonary circulation with their venous and arterial portions. The temporal effects of respiration on intrathoracic and abdominal pressures were integrated into the model according to Ursino and Magosso (2003).

The equations of the cardiovascular system may be found in the Supplementary Material (S1–S26), and the interested reader can find detailed information in Ursino and Magosso (2003) and Moscato et al. (2010, 2013).

#### Intrinsic Heart Rate Control

The intrinsic HR control was realized by a Hodgkin-Huxley-type single-cell human SA node model (Pohl et al., 2016). To cover the physiological range of HR, gating variables of the hyperpolarization-activated current, L-type Ca^2+^ current, acetylcholine (ACh)-activated K^+^ current, Na^+^/K^+^ pump current, slowly activating delayed rectifier K^+^ current, and sarcoendoplasmic reticulum Ca^2+^-ATPase (SERCA) activity were adjusted according to Fabbri et al. (2017). To simulate the effects of time-varying neurotransmitter concentrations, gating variable equations were further adjusted according to Zhang et al. (2002) and Li et al. (2014). Thus, the cell model incorporates the influence of the parasympathetic neurotransmitter ACh, and the sympathetic neurotransmitters epinephrine and norepinephrine using the β-sympathomimetic drug isoprenaline (Iso) as a surrogate. The modified model produced a depolarization rate of 74 bpm, which corresponds to the values reported in Fabbri et al. (2017) and is also in good accordance with the experimental findings of Verkerk et al. (2007), who report an experimentally determined spontaneous beating rate of 73 ± 3 bpm for isolated human SA node cells.

The cell model equations are given in the Supplementary Material (S57–S175). Detailed information on the development of the SA cell model may be found in Zhang et al. (2002); Li et al. (2014) and Fabbri et al. (2017).

The instantaneous HR is derived from the time differences between the peaks of consecutive SA node action potentials and fed to the hemodynamic model.

Finally, similar to Fabbri et al. (2017), not-null basal concentrations of Iso and ACh were manually determined so that the obtained resting HRs were in accordance with the values reported in the literature for HTxRs (McLaughlin et al., 1978; Crisafulli et al., 1985; Kavanagh et al., 1988; Labovitz et al., 1989; Wilson et al., 1991; Marzo et al., 1992; Rudas et al., 1993; Kao et al., 1994; Doering et al., 1996; Geny et al., 1996; Notarius et al., 1998; Hayman et al., 2010; Peled et al., 2017; Nygaard et al., 2019; Nytrøen et al., 2019) and age- and gender-matched healthy individuals (Crisafulli et al., 1985; Kavanagh et al., 1988; Wilson et al., 1991; Kao et al., 1994; Notarius et al., 1998; Hayman et al., 2010; Nygaard et al., 2019).

#### Autonomic Control

The autonomic control of total peripheral resistance, venous unstressed volume, left and right ventricular elastance, and HR was modeled according to Ursino and Magosso (2003). However, the proposed sigmoidal function to calculate the HR in the original model (Ursino and Magosso, 2003) was replaced by the previously described single-cell SA node model in which the depolarization rate is determined by time-varying neurotransmitter concentrations. Furthermore, inspired by the work of Magosso and Ursino (2002), additive terms were introduced in all effectors accounting for the influence of central command, which contributes significantly to the exercise response. Finally, the autonomic control model was extended by two linear equations, responsible for the modulation of aortic and arterial compliance based on exercise intensity. To implement the HR modulation by neurotransmitter released from sympathetic and parasympathetic cardiac nerve terminals, sympathetic outputs were linearly correlated with changes in Iso, and vagal outputs with changes in ACh concentrations. Finally, the calculated concentrations were added to the respective baseline values to find the total neurotransmitter concentrations.

The fundamental principle of exercise response was implemented according to the considerations of Moscato et al. (2013) and therein cited literature (Rowell, 1993), including the interaction of central command and muscle mechanoreflex and metaboreflex. Additionally, the release of circulating catecholamines by the adrenal medulla in response to physical activity was introduced into the model as a first-order dynamic system. The direct effects of the central command were modeled as previously described by Magosso and Ursino (2002).

Finally, a gradual exercise intensity-dependent decrease of the respiratory period was implemented, starting at a basal resting period of 5 s, eventually reaching 1.2 s at peak exercise. To simulate the muscle mechanoreflex and metaboreflex, peripheral resistance was decreased by 50% from an initial value of 0.97 mmHg⋅s/mL. The operating point of arterial baroreflex was increased by 90% from a basal value of 91 mmHg.

All equations governing the autonomic control can be found in the Supplementary Material (S16–S46).

#### Parameterization to Simulate HTxRs

Heart transplantation leads to severe changes in the autonomic feedback loops of HR and ventricular elastance control. This can be mainly attributed to efferent cardiac denervation impeding the vagal and sympathetic neural outflow to the allograft. On top of that, HTxRs also exhibit peripheral cardiovascular abnormalities, including increased total peripheral resistance and reduced vasodilatory capacity.

In Ursino and Magosso’s model of cardiac autonomic control (Ursino and Magosso, 2003), the magnitude of efferent outflow to the heart is determined by its cardiac effector gains. Therefore, to simulate cardiac denervation following HTx, modifications were applied to the gains of the heart period and ventricular elastance effectors. The vagal and sympathetic heart period effector gains were reduced by 100 and 95%, respectively. Furthermore, the sympathetic gain of ventricular elastance control was reduced by 95%.

To account for the peripheral changes in HTxRs, selected parameters of the hemodynamic model described by Moscato et al. (2010, 2013) were modified as follows. The total peripheral resistance was increased by 15%, whereas the peripheral compliance and the vasodilatory effect of muscle mechanoreflex and metaboreflex were decreased by 20 and 10%, respectively.

Possible changes in afterload resulting from HTx medication were implicitly incorporated into the model by tuning its parameters for pooled published data of HTxRs receiving typical post-HTx medication regimen (McLaughlin et al., 1978; Crisafulli et al., 1985; Kavanagh et al., 1988; Labovitz et al., 1989; Wilson et al., 1991; Marzo et al., 1992; Rudas et al., 1993; Kao et al., 1994; Doering et al., 1996; Geny et al., 1996; Notarius et al., 1998; Hayman et al., 2010; Peled et al., 2017; Nygaard et al., 2019; Nytrøen et al., 2019). A breakdown of the prescribed pharmacological agents by study can be found in Supplementary Table S1.

### Literature Data

#### Modeling Population

**FIGURE 2:**
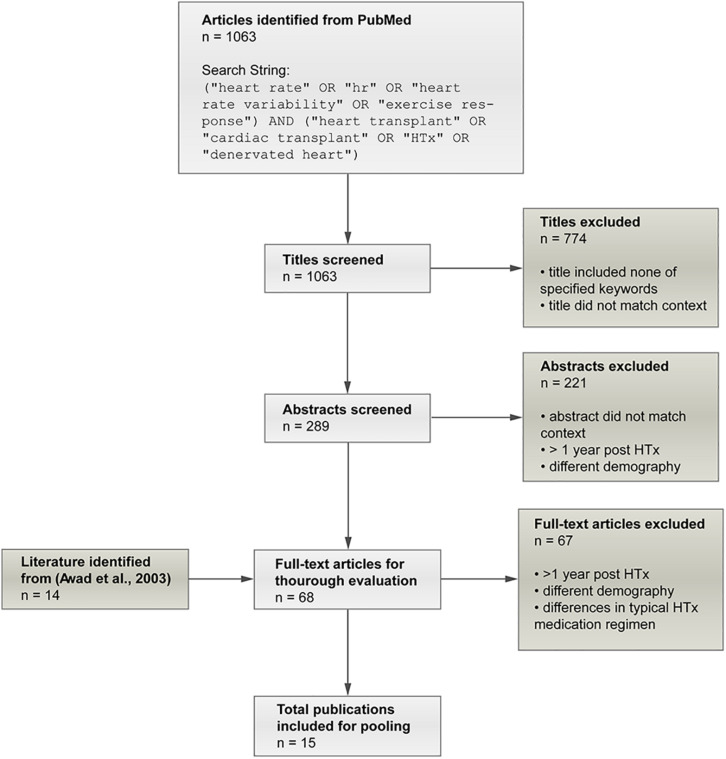
Summary of the screening process applied to identify eligible publications for data pooling. In the initial screening phase, publications were excluded if the title did reveal that the study includes hemodynamic variables neither for rest nor exercise and thus did not match the expected context. Finally, 15 publications (McLaughlin et al., 1978; Crisafulli et al., 1985; Kavanagh et al., 1988; Labovitz et al., 1989; Wilson et al., 1991; Marzo et al., 1992; Rudas et al., 1993; Kao et al., 1994; Doering et al., 1996; Geny et al., 1996; Notarius et al., 1998; Hayman et al., 2010; Peled et al., 2017; Nygaard et al., 2019; Nytrøen et al., 2019) could be identified as eligible for data pooling.

The literature review of Awad et al. (2016) on early denervation and later reinnervation in HTxRs served as the starting point for careful literature research to gather data for adjustment of the model to reproduce the hemodynamic parameters of HTxRs and age- and gender-matched healthy individuals. Initially, 14 publications (Pflugfelder et al., 1987; Smith et al., 1990; Schwaiger et al., 1991; Wilson et al., 1991, 1993, 2000; Kaye et al., 1993; Rudas et al., 1993; Burke et al., 1995; Doering et al., 1996, 1999; van De Borne et al., 2001; Toledo et al., 2002; Hayman et al., 2010) were identified as potential candidates for data pooling from Awad et al. (2016). After applying exclusion criteria, namely, different demographic characteristics, observation time longer than 1 year post-transplantation, and dissimilarities in the typical heart transplant medication regimen, four publications (Wilson et al., 1991; Rudas et al., 1993; Doering et al., 1996; Hayman et al., 2010) were identified from Awad et al. (2016), to be included for data pooling. Additionally, an online search on PubMed was conducted in January 2020, which yielded a total of 1,016 publications, and after applying the exclusion criteria, 11 eligible publications were identified (McLaughlin et al., 1978; Crisafulli et al., 1985; Kavanagh et al., 1988; Labovitz et al., 1989; Marzo et al., 1992; Kao et al., 1994; Geny et al., 1996; Notarius et al., 1998; Peled et al., 2017; Nygaard et al., 2019; Nytrøen et al., 2019). Therefore, a total of 15 publications (McLaughlin et al., 1978; Crisafulli et al., 1985; Kavanagh et al., 1988; Labovitz et al., 1989; Wilson et al., 1991; Marzo et al., 1992; Rudas et al., 1993; Kao et al., 1994; Doering et al., 1996; Geny et al., 1996; Notarius et al., 1998; Hayman et al., 2010; Peled et al., 2017; Nygaard et al., 2019; Nytrøen et al., 2019) were included in the pooling process. The careful literature screening process is summarized in Figure 2.

In all studies investigating exercise response (McLaughlin et al., 1978; Crisafulli et al., 1985; Kavanagh et al., 1988; Labovitz et al., 1989; Marzo et al., 1992; Kao et al., 1994; Notarius et al., 1998), modified square wave endurance exercise tests were performed in which the exercise intensity was increased until the maximum tolerated power (MTP) was reached. In one study (McLaughlin et al., 1978), the exercise test was performed in a supine position using an ergometer. In all other publications, the exercise tests were performed in upright positions, using either ergometers or treadmills.

Patients of all studies received a typical post-HTx medication regimen. In one publication (McLaughlin et al., 1978), no information on medication was given; therefore, we assumed conventional post-HTx medication as in the other studies. The breakdown of prescribed pharmacological agents by study can be found in Supplementary Table S1.

Finally, the pooled mean and variance of hemodynamic variables were calculated from a total of 15 publications (McLaughlin et al., 1978; Crisafulli et al., 1985; Kavanagh et al., 1988; Labovitz et al., 1989; Wilson et al., 1991; Marzo et al., 1992; Rudas et al., 1993; Kao et al., 1994; Doering et al., 1996; Geny et al., 1996; Notarius et al., 1998; Hayman et al., 2010; Peled et al., 2017; Nygaard et al., 2019; Nytrøen et al., 2019). If mean arterial pressure (MAP) was not given, it was calculated from systolic blood pressure (SBP) and diastolic blood pressure (DBP) by MAP = (SBP + 2 × DBP)/3. In six studies, cardiac output (CO) was calculated from VO_2_ using the regression equations suggested by Notarius et al. (1998).

In case that the body mass index was not given, it was calculated from height and weight. In one study (Labovitz et al., 1989), data were given as median and range; therefore, mean was substituted by the median, and variance was calculated dividing the given range by four.

The results for pooled hemodynamic parameters can be found in Table 1.

**TABLE 1:** Pooled means and variances of hemodynamic variables for HTxRs and matched healthy controls derived from literature compared to simulation results.

	HTxRs			Healthy		
	Rest^‡^					

Property	Mean ± Std.	*n*	Model	Mean ± Std.	*n*	Model
Resting HR (bpm)	92.7 ± 10.3	502	93.8	67.4 ± 10.8	178	69.5
Resting CO (L/min)^†^	5.9 ± 1.4	203	5.2	6.1 ± 1.2	113	5.6
Resting SBP (mmHg)	114.8 ± 27.4	339	111.3	119.7 ± 14.9	141	111.9
Resting DBP (mmHg)	75.9 ± 20.7	333	81.7	74.9 ± 10.3	141	74.2
Resting MAP (mmHg)^∗^	91.6 ± 21.4	387	91.5	91.5 ± 9.7	187	86.7
Resting SV (mL)	58.7 ± 15	384	55.4	86.2 ± 14.5	113	82
Resting SVR (mmHg⋅s/mL)^#^	0.96 ± 0.3	110	1.08	0.87 ± 0.16	68	0.97
Resting LVEDVI (mL/m^2^)	49.2 ± 12.1	110	51.9	65.7 ± 8.2	110	65.7

	**Exercise^§^**					

Peak HR (bpm)	129.3 ± 17.5	192	133.9	166.5 ± 14.9	97	169
Peak CO (L/min)^†^	12.9 ± 3.1	227	13	19.9 ± 3.6	97	19.8
Peak SBP (mmHg)	173 ± 22	54	161.3	211.5 ± 20.4	51	210.8
Peak DBP (mmHg)	97.4 ± 11.2	48	93.7	93.1 ± 11.9	51	95.2
Peak MAP (mmHg)^∗^	120.1 ± 15.9	109	116.3	131.2 ± 14.4	97	133.7
Peak SV (mL)^∥^	97.1 ± 19.7	186	95.7	120.7 ± 16.2	97	115.1
Peak SVR (mmHg⋅s/mL)^#^	0.62 ± 0.14	97	0.57	0.43 ± 0.07	82	0.45
Peak LVEDVI (mL/m^2^)^∗∗^	58.0 ± 11.6	30	66.1	67.3 ± 15.1	30	74.3
MTP (kpm/min)	523 ± 115	100	—	1,160 ± 212	90	—

*HR, heart rate; CO, cardiac output; SBP, systolic blood pressure; DBP, diastolic blood pressure; MAP, mean arterial pressure; SV, stroke volume; SVR, systemic vascular resistance; LVEDVI, left ventricular end-diastolic volume index; MTP, maximum tolerated power; *n*, number of pooled patients. ^∗^In eight publications (McLaughlin et al., 1978; Labovitz et al., 1989; Rudas et al., 1993; Kao et al., 1994; Nygaard et al., 2019), resting MAP, and in seven studies (McLaughlin et al., 1978; Labovitz et al., 1989; Rudas et al., 1993; Kao et al., 1994; Notarius et al., 1998; Nygaard et al., 2019), peak MAP was not given; therefore, it was approximated from SBP and DBP. ^†^In one publication (Marzo et al., 1992), resting CO, and in six publications (Crisafulli et al., 1985; Labovitz et al., 1989; Marzo et al., 1992; Notarius et al., 1998; Peled et al., 2017; Nytrøen et al., 2019) peak CO were calculated from VO_2_. ^∥^In eight publications (Crisafulli et al., 1985; Kavanagh et al., 1988; Labovitz et al., 1989; Wilson et al., 1991; Marzo et al., 1992; Kao et al., 1994; Geny et al., 1996; Notarius et al., 1998), peak SV was not given; therefore, it was calculated from CO and HR. ^#^For the control group in five studies (Crisafulli et al., 1985; Kavanagh et al., 1988; Marzo et al., 1992; Kao et al., 1994; Notarius et al., 1998) and for the HTxR group in four studies (Crisafulli et al., 1985; Kavanagh et al., 1988; Marzo et al., 1992; Kao et al., 1994), SVR was not given but could be calculated from CO and MAP. ^‡^Pooled data of HTxRs at rest from (McLaughlin et al., 1978; Crisafulli et al., 1985; Kavanagh et al., 1988; Labovitz et al., 1989; Marzo et al., 1992; Rudas et al., 1993; Kao et al., 1994; Doering et al., 1996; Geny et al., 1996; Notarius et al., 1998; Nygaard et al., 2019) and of healthy controls at rest from (Crisafulli et al., 1985; Kavanagh et al., 1988; Rudas et al., 1993; Kao et al., 1994; Doering et al., 1996; Notarius et al., 1998; Nygaard et al., 2019). ^§^Pooled data of HTxRs at peak exercise from (McLaughlin et al., 1978; Crisafulli et al., 1985; Labovitz et al., 1989; Marzo et al., 1992; Kao et al., 1994; Notarius et al., 1998) and of healthy controls for peak exercise from (Crisafulli et al., 1985; Kavanagh et al., 1988; Kao et al., 1994; Notarius et al., 1998). ^∗∗^Values of LVEDVI at peak exercise are for 37 and 71% of peak exercise intensities of HTxRs and the control group, respectively.*

#### Validation Population

For model validation, the simulation results for rest and peak exercise hemodynamics of HTxRs and healthy individuals were compared to published clinical data of an age- and gender-matched cohort other than that used for modeling but with comparable demographics (Pflugfelder et al., 1988; Hosenpud et al., 1989; Tamburino et al., 1989; Niset et al., 1991; Braith et al., 1992; Scott et al., 1995; Nytrøen et al., 2011; Cattermole et al., 2017) (Table 2). Because of a lack of data availability, the values used for validation of healthy resting hemodynamics were taken from a study reporting normative ranges of an age-matched healthy cohort (Cattermole et al., 2017). The literature was selected from an online search on Google Scholar, using the previously described set of keywords and exclusion criteria.

**TABLE 2:** Pooled means and variances of hemodynamic variables for HTxRs and an age- and gender-matched healthy control cohort compared to the simulation results for the purpose of model validation.

	HTxRs^‡^			Healthy^#∥^		
	Rest					

Property	Mean ± Std.	*n*	Model	Mean ± Std.	*n*	Model
Resting HR (bpm)	90.8 ± 11	253	93.8	71.3 ± 10.2	223	69.5
Resting CO (L/min)	5.5 ± 1	55	5.2	4.9 ± 1.3	223	5.6
Resting MAP (mmHg)	91.1 ± 10.7	55	91.5	94 ± 11.7	223	86.7
Resting SV (mL)	59.5 ± 9.9	55	55.4	68.8 ± 14.9	223	82
Resting SVR (mmHg⋅s/mL)	0.95 ± 0.23	145	1.08	1.17 ± 0.38	223	0.97
Resting LVEDVI (mL/m^2^)^†^	50 ± 15	22	51.9	—	—	65.7

	**Exercise**					

Peak HR (bpm)	127 ± 17	94	133.9	168 ± 15	10	169
Peak CO (L/min)	10.8 ± 4.3	32	13	20.8 ± 10	10	19.8
Peak MAP (mmHg)	105.1 ± 12.5	33	116.3	123 ± 9.5	10	133.7
Peak SV (mL)	87.8 ± 22.1	55	95.7	123 ± 35.6	10	115.1
Peak SVR (mmHg⋅s/mL)	0.61 ± 0.13	33	0.57	0.35 ± 0.13	10	0.45
Peak LVEDVI (mL/m^2^)^†^	59 ± 19	20	75.8	—	—	84.7
MTP (kpm/min)	584 ± 10	123	—	1,211 ± 288	10	—

*HR, heart rate; CO, cardiac output; SBP, systolic blood pressure; DBP, diastolic blood pressure; MAP, mean arterial pressure; SV, stroke volume; SVR, systemic vascular resistance; LVEDVI, left ventricular end-diastolic volume index; MTP, maximum tolerated power; n, number of pooled patients. ^†^Values for resting and peak LVEDVI in HTxRs were taken from Hosenpud et al. (1989). ^∥^Values for peak exercise in healthy individuals were taken from Braith et al. (1992). ^#^Values for resting condition in healthy individuals were taken from Cattermole et al. (2017). ^‡^Data for HTxRs at rest and exercise were pooled from Pflugfelder et al. (1988); Hosenpud et al. (1989), Tamburino et al. (1989); Niset et al. (1991), Scott et al. (1995), and Nytrøen et al. (2011).*

The results for pooled hemodynamic parameters can be found in Table 2.

### Simulation Protocols

Simulations were performed with model parameters adjusted for HTxRs and the healthy control group, both for rest and exercise conditions. The protocols were designed with two goals in mind. (1) The exercise protocol should be in accordance with the graded maximal exercise tests of the included studies (McLaughlin et al., 1978; Crisafulli et al., 1985; Kavanagh et al., 1988; Labovitz et al., 1989; Wilson et al., 1991; Marzo et al., 1992; Rudas et al., 1993; Kao et al., 1994; Doering et al., 1996; Geny et al., 1996; Notarius et al., 1998; Hayman et al., 2010; Peled et al., 2017; Nygaard et al., 2019; Nytrøen et al., 2019) and (2) the simulation duration should be sufficient to guarantee the stability of the model and therefore ensure the reliable determination of hemodynamic variables. Taking this into consideration, for resting condition, simulations of 300-s duration were performed. In the case of exercise simulations, the exercise intensities were increased in steps of 10% of maximum tolerable power until maximum exercise intensity was reached. As for resting condition, each exercise step was of 300-s duration. The simulated exercise intensity was adjusted so that peak CO complied with the pooled published data (McLaughlin et al., 1978; Crisafulli et al., 1985; Kavanagh et al., 1988; Labovitz et al., 1989; Wilson et al., 1991; Marzo et al., 1992; Rudas et al., 1993; Kao et al., 1994; Doering et al., 1996; Geny et al., 1996; Notarius et al., 1998; Hayman et al., 2010; Peled et al., 2017; Nygaard et al., 2019; Nytrøen et al., 2019). In HTxRs, exercise was terminated at 50% of the MTP of the control group, which is in accordance with the published clinical data (McLaughlin et al., 1978; Crisafulli et al., 1985; Kavanagh et al., 1988; Labovitz et al., 1989; Wilson et al., 1991; Marzo et al., 1992; Rudas et al., 1993; Kao et al., 1994; Doering et al., 1996; Geny et al., 1996; Notarius et al., 1998; Hayman et al., 2010; Peled et al., 2017; Nygaard et al., 2019; Nytrøen et al., 2019).

For both rest and exercise conditions, mean values of hemodynamic variables were calculated from time intervals of 300-s duration for resting condition, and each exercise step. HR was derived by averaging the reciprocal time differences between the peaks of consecutive SA node action potentials, MAP as the average of aortic pressure, mean DBP, and mean SBP from averaging minimum and maximum peak values of aortic pressures, respectively. CO was calculated as the average of left ventricular outflow, and stroke volume (SV) by averaging the differences between end-diastolic and end-systolic left ventricular volume. Systemic vascular resistance (SVR) was calculated as the average resistance of the systemic circulation compartment. Diastolic graft function was assessed through the end-diastolic volume index of the left ventricle (LVEDVI) and left ventricular end-diastolic pressure (LVEDP). The LVEDVI was computed by dividing the end-diastolic left ventricular volume by the mean body surface area (BSA) of the respective cohort. The BSA was calculated according to Gehan and George (1970).

## Results

**FIGURE 3:**
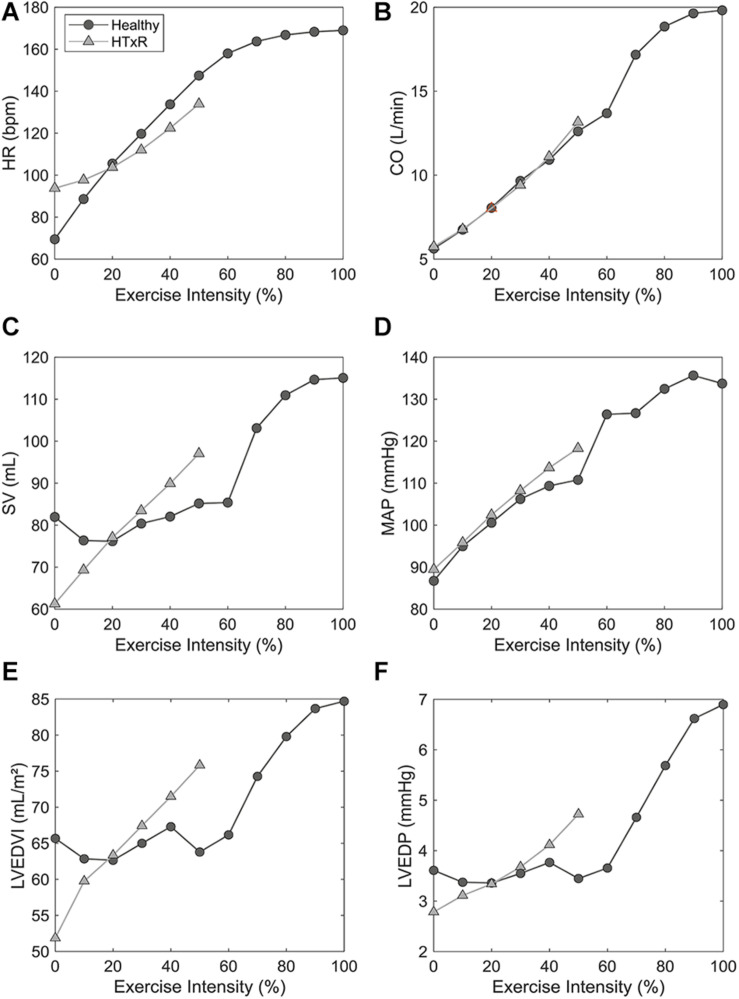
Comparison of selected hemodynamic variables derived from simulations to pooled published data (McLaughlin et al., 1978; Crisafulli et al., 1985; Kavanagh et al., 1988; Labovitz et al., 1989; Wilson et al., 1991; Marzo et al., 1992; Rudas et al., 1993; Kao et al., 1994; Doering et al., 1996; Geny et al., 1996; Notarius et al., 1998; Hayman et al., 2010; Peled et al., 2017; Nygaard et al., 2019; Nytrøen et al., 2019), for HTxRs and the healthy control group, both at rest and during exercise. HR, heart rate **(A)**; CO, cardiac output **(B)**; MAP, mean arterial pressure **(C)**; SVR, systemic vascular resistance **(D)**.

After tuning and optimization, the numerical model was found capable of producing hemodynamic parameters of early HTxRs and age- and gender-matched healthy cohorts (McLaughlin et al., 1978; Crisafulli et al., 1985; Kavanagh et al., 1988; Labovitz et al., 1989; Wilson et al., 1991; Marzo et al., 1992; Rudas et al., 1993; Kao et al., 1994; Doering et al., 1996; Geny et al., 1996; Notarius et al., 1998; Hayman et al., 2010; Peled et al., 2017; Nygaard et al., 2019; Nytrøen et al., 2019) with good accuracy. The simulation results for hemodynamic variables are reported in Table 1, whereas a comparison of selected hemodynamic variables is presented in Figure 3. Moreover, the model could predict HTxRs and normal hemodynamic values of a different study population (Pflugfelder et al., 1988; Hosenpud et al., 1989; Tamburino et al., 1989; Niset et al., 1991; Braith et al., 1992; Scott et al., 1995; Nytrøen et al., 2011; Cattermole et al., 2017), verifying the validity of the developed model. Ultimately, it shall be mentioned that the model does aim to reproduce the hemodynamics of a single patient but rather that of a whole cohort.

### Demographics

#### Modeling Population

The pooled demographics of HTxRs included age 48.8 ± 11.5 years, body mass index 27.2 ± 5 kg/m^2^, time post-HTx 3.8 ± 2.9 months, donor heart age 31.1 ± 11.4 years, and female gender 21.8%. If available, data from the publications were also pooled for demographically matched healthy controls (Crisafulli et al., 1985; Kavanagh et al., 1988; Wilson et al., 1991; Kao et al., 1994; Notarius et al., 1998; Hayman et al., 2010; Nygaard et al., 2019). The pooled demographics of healthy controls included age 46.6 ± 10.7 years, body mass index 25.5 ± 3 kg/m^2^, and female gender 23%.

#### Validation Population

The pooled demographics of HTxRs (Pflugfelder et al., 1988; Hosenpud et al., 1989; Tamburino et al., 1989; Niset et al., 1991; Scott et al., 1995; Nytrøen et al., 2011) included age 44.5 ± 11.2 years, body mass index 23 ± 3 kg/m^2^, time post-HTx 2.5 ± 0.6 months, donor heart age 31.2 ± 10.6 years, and female gender 13.9%. The pooled demographics of the healthy cohort used for validation of resting hemodynamics (Cattermole et al., 2017) included only age 45 ± 7.5 years, whereas the demographics of the healthy control group used for validation of exercise hemodynamics (Braith et al., 1992) included age, 50.4 ± 13.9 years, body mass index 26.9 ± 3.4 kg/m^2^, and female gender 9%.

### Exercise Capacity

With only 523 ± 115 kpm/min or 85 ± 19 W, the pooled published data (McLaughlin et al., 1978; Crisafulli et al., 1985; Kavanagh et al., 1988; Labovitz et al., 1989; Wilson et al., 1991; Marzo et al., 1992; Rudas et al., 1993; Kao et al., 1994; Doering et al., 1996; Geny et al., 1996; Notarius et al., 1998; Hayman et al., 2010; Peled et al., 2017; Nygaard et al., 2019; Nytrøen et al., 2019) show a marked reduction of nearly 50% in MTP of HTxRs as compared to the healthy control group with an MTP of 1,160 ± 212 kpm/min or 190 ± 35 W (Table 1). This is also reflected in the markedly reduced CO reaching only 12.9 ± 3.1 L/min in HTxRs (McLaughlin et al., 1978; Crisafulli et al., 1985; Kavanagh et al., 1988; Labovitz et al., 1989; Wilson et al., 1991; Marzo et al., 1992; Rudas et al., 1993; Kao et al., 1994; Doering et al., 1996; Geny et al., 1996; Notarius et al., 1998; Hayman et al., 2010; Peled et al., 2017; Nygaard et al., 2019; Nytrøen et al., 2019).

### Heart Rate

Simulations of resting condition predict baseline HR of 93.8 bpm (published data, 92.7 ± 10.3 bpm) for HTxRs. This equals an increase of about 25% compared to healthy baseline HR, which is consistent with the published data (Figure 3).

**FIGURE 4:**
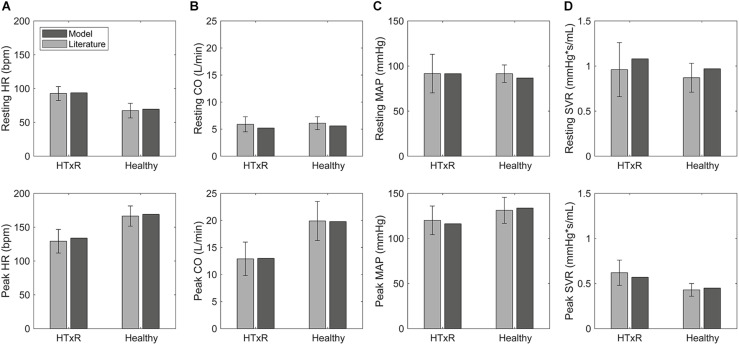
Comparison of exercise response between HTxRs and healthy controls, characterized by selected hemodynamic variables. The horizontal axis shows the relative exercise intensity with respect to the peak exercise intensity of the healthy control group (1,032 kpm/min). Note the substantial reduction of exercise capacity in HTxRs, reaching only about 50% (522 kpm/min) of the healthy control’s peak exercise intensity. We can see a marked impairment of chronotropic response and reduced peak HR in HTxRs **(A)**. Furthermore, the simulation results show no significant differences in augmentation in CO but substantially decreased peak CO in HTxRs **(B)**. Resting SV is notably reduced in HTxRs and undergoes a rapid increase in response to exercise **(C)**. MAP response is similar in both groups; however, healthy controls reach higher peak values due to greater exercise intensity **(D)**. Despite the reduced absolute values, HTxRs show a markedly stronger augmentation of LVEDVI in response to exercise **(E)**. Simulations predict a similar resting LVEDP for both the HTxRs, however, the augmentation of LVEDP is markedly stronger than in age- and gender-matched healthy individuals **(F)**. HR, heart rate; CO, cardiac output; SV, stroke volume; MAP, mean arterial pressure; LVEDVI, left ventricular end-diastolic volume index; LVEDP, left ventricular end-diastolic pressure.

Especially noticeable is the reduced chronotropic response in HTxRs. The model predicts a peak HR of only 133.9 bpm (published data, 129.3 ± 17.5 bpm), which represents a reduction of more than 20% compared to the healthy control group (Figure 3). We can clearly see the impaired chronotropic response in HTxRs, which is characterized by the notably lower slope of the curve compared to the control group (Figure 4A).

### Stroke Volume

In contrast to the healthy control group, simulations show a marked reduction in resting SV by 25% to only 55.4 mL (published data, 58.7 ± 15 mL) in HTxRs.

In response to exercise, simulations of HTxRs show strong SV augmentation, eventually reaching a maximum value of 95.7 mL (published data, 97.1 ± 19.7 mL), which is about 20% lower than in the healthy individuals (Figure 3).

The model predicts notably stronger SV augmentation in HTxRs than in age- and gender-matched healthy controls, although reaching subnormal values at their respective peak exercise (Figure 4C).

### Cardiac Output

Simulations of resting condition show normal basal CO of 5.2 L/min (published data, 5.9 ± 1.4 L/min) for HTxRs comparable to results for the healthy control group (Figure 3).

However, exercise simulations of HTxRs predict a peak CO of only 13 L/min (published data, 12.9 ± 3.1 L/min), which represents a drastic reduction of more than 40%, compared to healthy individuals (Figure 3). Despite the reduced peak CO, in response to low- to moderate-intensity exercise, the model predicts an almost identical increase of CO in both groups (Figure 4B). Differences become clear only at moderate- to high-intensity exercise for which healthy persons can further increase their CO, eventually reaching nearly twice the values of HTxRs (Figure 4B).

### Left Ventricular End-Diastolic Volume

Consistent with the pooled published data, simulations of HTxRs at rest predict an LVEDVI of 51.9 mL/m^2^ (published data, 49.2 ± 12.1 mL/m^2^), which is about 20% lower than that of age- and gender-matched healthy individuals (Table 1).

In contrast to the control group, simulations of exercise in HTxRs show a strong and immediate augmentation of LVEDVI, reaching 75.8 mL/m^2^ at their respective peak exercise load, which is about 15% higher than that of healthy individuals at the same exercise intensity (Figure 4E).

Despite HTxRs show a stronger relative increase in LVEDVI, the model predicts notably higher total LVEDVI of 84.7 mL/m^2^ at peak exercise in the control group.

However, only one publication (Kao et al., 1994) of the literature used for data pooling included values for LVEDVI at peak exercise. In this study, the peak exercise load was 390 and 825 kpm/min for HTxRs and the control group, respectively, which equals an exercise intensity of 37 and 71%, respectively, in our simulation. For these intensities, the model predicts an LVEDVI of 66.1 mL/m^2^ (published data, 58 ± 11.6 mL/m^2^) for HTxRs and 75.3 mL/m^2^ (published data, 67.3 ± 15.1 mL/m^2^) for age- and gender-matched healthy individuals, which is in good accordance with the values published in the literature (Kao et al., 1994).

### Left Ventricular End-Diastolic Pressure

Simulations predict a similar resting LVEDP for both the HTxRs and the age- and gender-matched healthy control group with 2.8 and 3.6 mmHg, respectively (Figure 4F).

In HTxRs, the augmentation of LVEDP is markedly stronger than in age- and gender-matched healthy individuals (Figure 4F). HTxRs reach about 4.4 mmHg at peak exercise, which is about 20% higher than that of healthy individuals at the same exercise intensity. However, the LVEDP of 6.9 mmHg reached by healthy individuals at peak exercise is 30% higher than that of HTxRs at their respective peak exercise intensity.

None of the publications included for data pooling reported values of LVEDP at rest or exercise.

### Model Validation

Except for the peak exercise MAP of healthy individuals, the simulation results are in good accordance with the pooled literature data, all being within the standard deviation of patient variability. Although the predicted peak exercise MAP is not in this range, that does not necessarily indicate a bad model representation of exercise hemodynamics. The small discrepancy between predicted and true MAP is consistent with the comparably lower SVR of the healthy study cohort (Braith et al., 1992) (Table 2). Overall, the simulation results are consistent with published clinical data (Pflugfelder et al., 1988; Hosenpud et al., 1989; Tamburino et al., 1989; Niset et al., 1991; Braith et al., 1992; Scott et al., 1995; Nytrøen et al., 2011; Cattermole et al., 2017), therefore verifying the validity of the presented model.

## Discussion

The model predictions for cohort hemodynamics of early HTxRs and age- and gender-matched healthy individuals are in good accordance with pooled published data (Doering et al., 1996; Wilson et al., 1991; Hayman et al., 2010; Rudas et al., 1993; Geny et al., 1996; Kao et al., 1994; Nygaard et al., 2019; Kavanagh et al., 1988; Marzo et al., 1992; Peled et al., 2017; McLaughlin et al., 1978; Labovitz et al., 1989; Notarius et al., 1998; Crisafulli et al., 1985; Nytrøen et al., 2019; Pflugfelder et al., 1988; Scott et al., 1995; Tamburino et al., 1989; Niset et al., 1991; Nytrøen et al., 2011; Hosenpud et al., 1989; Braith et al., 1992; Cattermole et al., 2017), both for rest and exercise (Figure 3). The simulations could provide important insights on hemodynamic changes in early HTxRs.

Simulations predict that the markedly increased resting HR in early HTxRs is exclusively due to the absence of vagal tone resulting from parasympathetic cardiac denervation and that, supersensitivity to circulating catecholamines, is neglectable. However, normal CO is still maintained through a reduction of SV by about 25% compared to age- and gender-matched healthy individuals (Figure 3).

Heart transplantation recipients suffer from a notably reduced exercise capacity (McLaughlin et al., 1978; Crisafulli et al., 1985; Kavanagh et al., 1988; Labovitz et al., 1989; Wilson et al., 1991; Marzo et al., 1992; Rudas et al., 1993; Kao et al., 1994; Doering et al., 1996; Geny et al., 1996; Notarius et al., 1998; Hayman et al., 2010; Awad et al., 2016; Kobashigawa and Olymbios, 2017; Peled et al., 2017; Nygaard et al., 2019; Nytrøen et al., 2019), yet still, there is no general agreement to which extent the limiting factors are of cardiopulmonary or peripheral nature (Awad et al., 2016; Kobashigawa and Olymbios, 2017).

Simulations of maximum graded exercise do not show abnormally elevated pulmonary pressure in HTxRs at any intensity. HTxRs do reach a left atrial pressure (LAP) of 11 mmHg at peak exercise, which is similar to that of healthy controls, which attain a LAP of 11.6 mmHg at 50% exercise intensity, suggesting that reduced exercise capacity does not result from early dyspnea. However, for the sake of brevity, the results of the LAP response are not presented here.

The model predicts a strongly impeded chronotropic response in HTxRs, reaching only about 80% of the normal peak HR (Figure 3). The reduced HR response at low exercise intensities (up to 20%) results from a lack of vagal withdrawal due to complete vagal cardiac denervation. The inability to sufficiently increase the HR in response to exercise intensities greater than 30% is a consequence of the virtually absent sympathetic drive that is reduced by 95% due to sympathetic cardiac denervation. The remaining HR augmentation primarily relies on circulating catecholamines, which is also general consent in literature (Awad et al., 2016; Kobashigawa and Olymbios, 2017).

Ion-channel gating mechanisms of the SA node and β-agonist clearing remained unchanged in HTxRs. Therefore, the model neglects the often assumed supersensitivity of the donor heart to circulating catecholamines (Awad et al., 2016; Kobashigawa and Olymbios, 2017). Nevertheless, model predictions for exercise response still are in excellent accordance with literature data, suggesting that supersensitivity may play a neglectable role in exercise response compensation in early HTxRs. A possible explanation is that supersensitivity typically tends to develop over time (Kobashigawa and Olymbios, 2017), thus being not present yet in the modeled population with a post-HTx time of 3.8 ± 2.9 months.

Moreover, simulations attain 20% less vasodilation in HTxRs (Figure 3), contributing to the limitation in CO augmentation. Impaired muscular vasodilation may be the result of prolonged periods of deconditioning pre-HTx (Kobashigawa and Olymbios, 2017) and changes in the vasopressor effect associated with cyclosporine treatment (Andreassen et al., 1998).

**FIGURE 5:**
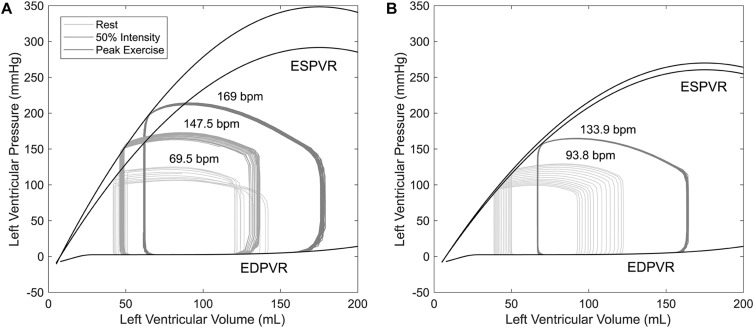
Pressure–volume loops for a time period of 10 s obtained from simulations of healthy controls **(A)** and HTxRs **(B)**, for rest (0%), medium (50%), and peak (100%) exercise intensity. Note that peak exercise in HTxRs corresponds to 50% of exercise intensity in healthy individuals.

For low- to medium-intensity exercise, healthy individuals mainly rely on HR increase through vagal withdrawal to augment their CO (Figure 4), whereas enhanced ventricular contractility plays a minor role (Figure 5A). However, for medium- to high-intensity exercise, healthy individuals further increase their CO primarily through SV augmentation, while onward HR augmentation plays a secondary role (Figure 5A). The increase in SV can be attributed to raised contractility, characterized by the increased slope of the end-systolic pressure–volume relationship and increasing end-diastolic volume through the Frank–Starling mechanism (Figure 5A).

Simulations show that despite the reduced exercise capacity, for low to moderate intensity, HTxRs can maintain normal CO, which is consistent with the findings in the literature (Kobashigawa and Olymbios, 2017). However, in contrast to healthy individuals, the exercise response in HTxRs is quite different. The model predicts that for any given exercise intensity, HTxRs strongly rely on the increase of preload through the Frank–Starling mechanism, enhancing their SV to compensate for chronotropic and inotropic incompetence (Figure 5B). Nevertheless, an increase in HR through circulating catecholamines plays a non-neglectable, yet secondary role for CO augmentation (Figure 4).

Diastolic dysfunction is an often-reported condition in HTxRs manifesting in a leftward shift of the end-diastolic pressure-volume relationship (EDPVR); however, the underlying mechanisms are still not entirely understood. Possible reasons include cardiac denervation, heart graft remodeling, ischemia, and graft rejection (Rudas et al., 1990). In our model, we did not explicitly modify the EDPVR, thus not accounting for possible diastolic graft dysfunction. Consequently, the cardiac model represents a properly vascularized heart graft early after transplantation in which no considerable cardiac remodeling has taken place and that is not affected by graft rejection, which reflects the modeled population of early HTxRs (McLaughlin et al., 1978; Crisafulli et al., 1985; Kavanagh et al., 1988; Labovitz et al., 1989; Wilson et al., 1991; Marzo et al., 1992; Rudas et al., 1993; Kao et al., 1994; Doering et al., 1996; Geny et al., 1996; Notarius et al., 1998; Hayman et al., 2010; Peled et al., 2017; Nygaard et al., 2019; Nytrøen et al., 2019). Simulations to investigate the diastolic graft dysfunction and its influence on resting and especially on exercise hemodynamics should be the focus of future studies.

Still, the model predicts evident acute changes in diastolic pressures and volumes in early HTxRs, showing markedly reduced resting and peak LVEDVI compared to the control group. The values for LVEDVI at rest and exercise are in good accordance with the literature (Kao et al., 1994; Nygaard et al., 2019), suggesting that despite the reduced absolute values, HTxRs show a markedly stronger augmentation of LVEDVI in response to exercise (Figure 4E). Ultimately, the simulations predict similar LVEDP at rest in both groups, while the pressure increases notably stronger with exercise load in HTxRs than in the control group, allowing a greater increase in LVEDV.

The model was validated based on published clinical data of patients other than used for tuning of the model. For HTxRs, all predicted hemodynamic values are within the range of one standard deviation of the true values (Table 2), therefore suggesting a good model representation of resting and exercise hemodynamics in early HTxRs. Except for peak exercise MAP, the model predictions for the healthy group are also in good accordance with the published clinical data. The predicted peak exercise MAP is not in the range of one standard deviation of natural interpatient variability; however, the trend in the MAP response to exercise is the same as in the HTxR group. The small discrepancy in MAP is also consistent with the differences between predicted and true SVR (Table 2). The sample size of the cohort used to validate the model of normal exercise hemodynamics is rather small (*n* = 10); therefore, it might be necessary to further validate the model based on hemodynamic data of a larger group.

Summarizing, according to the simulation results, in early HTxRs, the limited exercise response is primarily due to virtually absent sympathetic drive, which is reduced by 95% due to sympathetic cardiac denervation. The simulations highlight chronotropic incompetence following sympathetic cardiac denervation as the main limiting factor for exercise in HTxRs. Peripheral factors seem to play a secondary role, while pulmonary factors are negligible in the limitation of exercise capacity. The model shows that the main compensatory mechanism can be attributed to SV augmentation strongly relying on increased preload, while supersensitivity to circulating catecholamines is insignificant in early HTxRs.

### Limitations of the Study

The model was validated with a small complementary set of clinical hemodynamic data of HTxRs and healthy individuals. However, the data contained only a limited number of patient measurements and hemodynamic parameters. Consequently, the model should undergo further validation, based on a more comprehensive dataset.

The often-observed diastolic dysfunction in HTxRs was not explicitly modeled. Thus, the cardiac model represents a properly vascularized, short-term post-HTx condition in which no remodeling has taken place yet. Simulations to investigate the diastolic graft dysfunction and its influence on resting and exercise hemodynamics were not part of this study but certainly interesting for future study, to investigate late HTxR hemodynamic response.

## Conclusion

The present study provides a model-based perspective on the hypothesized origins of reduced exercise capacity and compensatory mechanisms for chronotropic and inotropic incompetence in early HTxRs.

Simulation results show an overall reduced exercise capacity in early HTxRs, which is primarily due to chronotropic incompetence, whereas peripheral factors play a secondary role. HTxRs can maintain normal CO for low- to medium-intensity exercise by compensation of chronotropic and inotropic incompetence through increased filling pressures.

## Data Availability Statement

The raw data supporting the conclusions of this article will be made available by the authors, without undue reservation.

## Author Contributions

MH and FM contributed to the conception and design of the study and were involved in the interpretation of the study results. MH and DD conducted the literature review, contributed to the model development, and performed the simulations. DD performed the data pooling. MH wrote the manuscript and generated the illustrations. All authors contributed to manuscript revision, read, and approved the submitted version.

## Conflict of Interest

The authors declare that the research was conducted in the absence of any commercial or financial relationships that could be construed as a potential conflict of interest.

## References

[B1] Andreassen A. K. Kvernebo K. Jørgensen B. Simonsen S. Kjekshus J. Gullestad L. (1998). Exercise capacity in heart transplant recipients: relation to impaired endothelium-dependent vasodilation of the peripheral microcirculation. *Am. Heart J.* 136 320–328. 10.1053/hj.1998.v136.89731 9704697

[B2] Awad M. Czer L. S. Hou M. Golshani S. S. Goltche M. De Robertis M. (2016). Early denervation and later reinnervation of the heart following cardiac transplantation: a review. *J. Am. Heart Assoc.* 5:e004070. 10.1161/JAHA.116.004070 27802930 PMC5210323

[B3] Banerjee R. K. Ashtekar K. D. Helmy T. A. (2008). Hemodynamic diagnostics of epicardial coronary stenoses: in-vitro experimental and computational study. *Biomed. Eng. Online* 7:24. 10.1186/1475-925X-7-24 18752683 PMC2556321

[B4] Braith R. W. Wood C. E. Limacher M. C. Pollock M. L. Lowenthal D. T. Phillips M. I. (1992). Abnormal neuroendocrine responses during exercise in heart transplant recipients. *Circulation* 86 1453–1463. 10.1161/01.cir.86.5.1453 1423959

[B5] Burke M. N. McGinn A. L. Homans D. C. Christensen B. V. Kubo S. H. Wilson R. F. (1995). Evidence for functional sympathetic reinnervation of left ventricle and coronary arteries after orthotopic cardiac transplantation in humans. *Circulation* 91 72–78. 7646628 10.1161/01.cir.91.1.72

[B6] Cattermole G. N. Leung P. Y. M. Ho G. Y. L. Lau P. W. S. Chan C. P. Y. Chan S. S. W. (2017). The normal ranges of cardiovascular parameters measured using the ultrasonic cardiac output monitor. *Physiol. Rep.* 5:e13195. 10.14814/phy2.13195 28320891 PMC5371563

[B7] Crisafulli A. Tocco F. Milia R. Angius L. Pinna M. Olla S. (1985). Progressive improvement in hemodynamic response to muscle metaboreflex in heart transplant recipients. *J. Appl. Physiol.* 114 421–427. 10.1152/japplphysiol.01099.2012 23195627

[B8] Doering L. V. Dracup K. Moser D. K. Czer L. S. Peter C. T. (1996). Hemodynamic adaptation to orthostatic stress after orthotopic heart transplantation. *Heart Lung* 25 339–351. 10.1016/S0147-9563(96)80076-8 8886810

[B9] Doering L. V. Dracup K. Moser D. K. Czer L. S. Peter C. T. (1999). Evidence of time-dependent autonomic reinnervation after heart transplantation. *Nurs. Res.* 48 308–316. 10.1097/00006199-199911000-00006 10571498

[B10] Fabbri A. Fantini M. Wilders R. Severi S. (2017). Computational analysis of the human sinus node action potential: model development and effects of mutations. *J. Physiol.* 595 2365–2396. 10.1113/jp273259 28185290 PMC5374121

[B11] Gehan E. A. George S. L. (1970). Estimation of human body surface area from height and weight 12. *Cancer Chemother. Rep.* 54 225–235. 5527019

[B12] Geny B. Saini J. Mettauer B. Lampert E. Piquard F. Follenius M. (1996). Effect of short-term endurance training on exercise capacity, haemodynamics and atrial natriuretic peptide secretion in heart transplant recipients. *Eur. J. Appl. Physiol. Occup. Physiol.* 73 259–266. 10.1007/BF02425485 8781855

[B13] Gross C. Moscato F. Schlöglhofer T. Maw M. Meyns B. Marko C. (2020). LVAD speed increase during exercise, which patients would benefit the most? A simulation study. *Artif. Organs* 44 239–247. 10.1111/aor.13569 31519043

[B14] Hayman M. A. Nativi J. N. Stehlik J. McDaniel J. Fjeldstad A. S. Ives S. J. (2010). Understanding exercise-induced hyperemia: central and peripheral hemodynamic responses to passive limb movement in heart transplant recipients. *Am. J. Physiol. Heart Circ. Physiol.* 299 1653–1659. 10.1152/ajpheart.00580.2010 20833963 PMC2993203

[B15] Hosenpud J. D. Morton M. J. Wilson R. A. Pantely G. A. Norman D. J. Cobanoglu M. A. (1989). Abnormal exercise hemodynamics in cardiac allograft recipients 1 year after cardiac transplantation. Relation to preload reserve. *Circulation* 80 525–532. 10.1161/01.cir.80.3.525 2670315

[B16] Jung J. Lyczkowski R. W. Panchal C. B. Hassanein A. (2006). Multiphase hemodynamic simulation of pulsatile flow in a coronary artery. *J. Biomech.* 39 2064–2073. 10.1016/j.jbiomech.2005.06.023 16111686

[B17] Kao A. C. Van Trigt P. III Shaeffer-McCall G. S. Shaw J. P. Kuzil B. B. Page R. D. (1994). Central and peripheral limitations to upright exercise in untrained cardiac transplant recipients. *Circulation* 89 2605–2615. 10.1161/01.CIR.89.6.2605 8205672

[B18] Katona P. G. Poitras J. W. Barnett G. O. Terry B. S. (1970). Cardiac vagal efferent activity and heart period in the carotid sinus reflex. *Am. J. Physiol. Leg. Content* 218 1030–1037. 10.1152/ajplegacy.1970.218.4.1030 5435400

[B19] Kavanagh T. Yacoub M. H. Mertens D. J. Kennedy J. Campbell R. B. Sawyer P. (1988). Cardiorespiratory responses to exercise training after orthotopic cardiac transplantation. *Circulation* 77 162–171. 10.1161/01.cir.77.1.162 3275506

[B20] Kaye D. M. Esler M. Kingwell B. McPherson G. Esmore D. Jennings G. (1993). Functional and neurochemical evidence for partial cardiac sympathetic reinnervation after cardiac transplantation in humans. *Circulation* 88 1110–1118. 10.1161/01.CIR.88.3.1110 8353872

[B21] Kember G. Ardell J. L. Armour J. A. Zamir M. (2014). Vagal nerve stimulation therapy: what is being stimulated? *PLoS One* 9:e114498. 10.1371/journal.pone.0114498 25479368 PMC4257685

[B22] Kember G. Ardell J. L. Shivkumar K. Armour J. A. (2017). Recurrent myocardial infarction: mechanisms of free-floating adaptation and autonomic derangement in networked cardiac neural control. *PLoS One* 12:e0180194. 10.1371/journal.pone.0180194 28692680 PMC5503241

[B23] Khush K. K. Cherikh W. S. Chambers D. C. Harhay M. O. Hayes D. Hsich E. (2019). The international thoracic organ transplant registry of the international society for heart and lung transplantation: thirty-sixth adult heart transplantation report - 2019; focus theme: donor and recipient size match. *J. Heart Lung Transplant.* 38 1056–1066. 10.1016/j.healun.2019.08.004 31548031 PMC6816343

[B24] Kobashigawa J. Olymbios M. (2017). “Physiology of the transplanted heart,” in *Clinical Guide to Heart Transplantation*, ed. Kobashigawa J. (Cham: Springer), 81–93. 10.1007/978-3-319-43773-6_8

[B25] Labovitz A. J. Drimmer A. M. McBride L. R. Pennington D. G. Willman V. L. Miller L. W. (1989). Exercise capacity during the first year after cardiac transplantation. *Am. J. Cardiol.* 64 642–645. 10.1016/0002-9149(89)90494-3 2675585

[B26] Levy M. N. Zieske H. (1969). Autonomic control of cardiac pacemaker activity and atrioventricular transmission. *J. Appl. Physiol.* 27 465–470. 10.1152/jappl.1969.27.4.465 5822553

[B27] Li X. Zhang J. Shuai J. (2014). Isoprenaline: a potential contributor in sick sinus syndrome—insights from a mathematical model of the rabbit sinoatrial node. *Sci. World J.* 67 1725–1738. 10.1155/2014/540496 24578642 PMC3918845

[B28] Magosso E. Ursino M. (2002). Cardiovascular response to dynamic aerobic exercise: a mathematical model. *Med. Biol. Eng. Comput.* 40 660–674. 10.1007/BF02345305 12507317

[B29] Marzo K. P. Wilson J. R. Mancini D. M. (1992). Effects of cardiac transplantation on ventilatory response to exercise. *Am. J. Cardiol.* 69 547–553. 10.1016/0002-9149(92)91002-L 1736622

[B30] McLaughlin P. R. Kleiman J. H. Martin R. P. Doherty P. W. Reitz B. Stinson E. B. (1978). The effect of exercise and atrial pacing on left ventricular volume and contractility in patients with innervated and denervated hearts. *Circulation* 58 476–483. 10.1161/01.CIR.58.3.476 679438

[B31] Moscato F. Arabia M. Colacino F. M. Naiyanetr P. Danieli G. A. Schima H. (2010). Left ventricle afterload impedance control by an axial flow ventricular assist device: a potential tool for ventricular recovery. *Artif. Organs* 34 736–744. 10.1111/j.1525-1594.2010.01066.x 20636446

[B32] Moscato F. Wirrmann C. Granegger M. Eskandary F. Zimpfer D. Schima H. (2013). Use of continuous flow ventricular assist devices in patients with heart failure and a normal ejection fraction: a computer-simulation study. *J. Thorac. Cardiovasc. Surg.* 145 1352–1358. 10.1016/j.jtcvs.2012.06.057 22841169

[B33] Niset G. Hermans L. Depelchin P. (1991). Exercise and heart transplantation. *Sports Med.* 12 359–379. 10.2165/00007256-199112060-00003 1784879

[B34] Notarius C. F. Levy R. D. Tully A. Fitchett D. Magder S. (1998). Cardiac versus noncardiac limits to exercise after heart transplantation. *Am. Heart J.* 135 339–348. 10.1016/S0002-8703(98)70103-6 9489986

[B35] Nygaard S. Christensen A. H. Rolid K. Nytrøen K. Gullestad L. Fiane A. (2019). Autonomic cardiovascular control changes in recent heart transplant recipients lead to physiological limitations in response to orthostatic challenge and isometric exercise. *Eur. J. Appl. Physiol.* 119 2225–2236. 10.1007/s00421-019-04207-5 31407088 PMC6763412

[B36] Nytrøen K. Myers J. Chan K. N. Geiran O. R. Gullestad L. (2011). Chronotropic responses to exercise in heart transplant recipients. *Am. J. Phys. Med. Rehabil.* 90 579–588. 10.1097/phm.0b013e31821f711d 21765276

[B37] Nytrøen K. Rolid K. Andreassen A. K. Yardley M. Gude E. Dahle D. O. (2019). Effect of high-intensity interval training in de novo heart transplant recipients in Scandinavia. *Circulation* 139 2198–2211. 10.1161/CIRCULATIONAHA.118.036747 30773030

[B38] Okwuosa I. S. Lewsey S. C. Adesiyun T. Blumenthal R. S. Yancy C. W. (2016). Worldwide disparities in cardiovascular disease: challenges and solutions. *Int. J. Cardiol.* 202 433–440. 10.1016/j.ijcard.2015.08.172 26433167

[B39] Peled Y. Varnado S. Lowes B. D. Zolty R. Lyden E. R. Moulton M. J. (2017). Sinus tachycardia is associated with impaired exercise tolerance following heart transplantation. *Clin. Transplant.* 31:e12946. 10.1111/ctr.12946 28251691

[B40] Pflugfelder P. W. McKenzie F. N. Kostuk W. J. (1988). Hemodynamic profiles at rest and during supine exercise after orthotopic cardiac transplantation. *Am. J. Cardiol.* 61 1328–1333. 10.1016/0002-9149(88)91178-2 3287883

[B41] Pflugfelder P. W. Purves P. D. McKenzie F. N. Kostuk W. J. (1987). Cardiac dynamics during supine exercise in cyclosporine-treated orthotopic heart transplant recipients: assessment by radionuclide angiography. *J. Am. Coll. Cardiol.* 10 336–341. 10.1016/S0735-1097(87)80016-5 3298362

[B42] Pohl A. Wachter A. Hatam N. Leonhardt S. (2016). A computational model of a human single sinoatrial node cell. *Biomed. Phys. Eng. Express* 2:035006 10.1088/2057-1976/2/3/035006 37608504

[B43] Politi M. T. Ghigo A. Fernández J. M. Khelifa I. Gaudric J. Fullana J. M. (2016). The dicrotic notch analyzed by a numerical model. *Comput. Biol. Med.* 72 54–64. 10.1016/j.compbiomed.2016.03.005 27016670

[B44] Qian Y. Liu J. L. Itatani K. (2010). Computational hemodynamic analysis in congenital heart disease: simulation of the Norwood procedure. *Ann. Biomed. Eng.* 38 2302–2313. 10.1007/s10439-010-9978-5 20195758

[B45] Rowell L. B. (1993). *Human Cardiovascular Control.* New York, NY: Oxford University Press 10.1002/clc.4960170212

[B46] Rudas L. Pflugfelder P. W. Kostuk W. J. (1990). Comparison of hemodynamic responses during dynamic exercise in the upright and supine postures after orthotopic cardiac transplantation. *J. Am. Coll. Cardiol.* 16 1367–1373. 10.1016/0735-1097(90)90378-3 2229788

[B47] Rudas L. Pflugfelder P. W. Kostuk W. J. (1993). Immediate cardiovascular responses to orthostasis in the early and late months after cardiac transplantation. *Int. J. Cardiol.* 38 141–150. 10.1016/0167-5273(93)90173-E 8454376

[B48] Schwaiger M. Hutchins G. D. Kalff V. Rosenspire K. Haka M. S. Mallette S. (1991). Evidence for regional catecholamine uptake and storage sites in the transplanted human heart by positron emission tomography. *J. Clin. Invest.* 87 1681–1690. 10.1172/JCI115185 2022739 PMC295266

[B49] Scott C. D. Dark J. H. McComb J. M. (1995). Evolution of the chronotropic response to exercise after cardiac transplantation. *Am. J. Cardiol.* 76 1292–1296. 10.1016/s0002-9149(99)80358-0 7503012

[B50] Smith M. L. Ellenbogen K. A. Eckberg D. L. Sheehan H. M. Thames M. D. (1990). Subnormal parasympathetic activity after cardiac transplantation. *Am. J. Cardiol.* 66 1243–1246. 10.1016/0002-9149(90)91108-i 2239730

[B51] Tamburino C. Corcos T. Feraco E. Leger P. Desruennes M. Vaissier E. (1989). Hemodynamic parameters one and four weeks after cardiac transplantation. *Am. J. Cardiol.* 63 635–637. 10.1016/0002-9149(89)90917-x 2645764

[B52] Timmis A. Townsend N. Gale C. P. Torbica A. Lettino M. Petersen S. E. (2020). European society of cardiology: cardiovascular disease statistics 2019. *Eur. Heart J.* 41 12–85. 10.1093/eurheartj/ehz859 31820000

[B53] Toledo E. Pinhas I. Aravot D. Almog Y. Akselrod S. (2002). Functional restitution of cardiac control in heart transplant patients. *Am. J. Physiol. Regul. Integr. Comp. Physiol.* 282 R900–R908. 10.1152/ajpregu.00467.2001 11832413

[B54] Ursino M. Magosso E. (2003). Role of short-term cardiovascular regulation in heart period variability: a modeling study. *Am. J. Physiol. Heart Circ. Physiol.* 284 1479–1493. 10.1152/ajpheart.00850.2002 12595291

[B55] van De Borne P. Neubauer J. Rahnama M. Jansens J. L. Montano N. Porta A. (2001). Differential characteristics of neural circulatory control: early versus late after cardiac transplantation. *Circulation* 104 1809–1813. 10.1161/hc4101.097248 11591619

[B56] Verkerk A. O. van Borren M. M. Peters R. J. Broekhuis E. Lam K. Y. Coronel R. (2007). “Single cells isolated from human sinoatrial node: action potentials and numerical reconstruction of pacemaker current,” in *Proceedings of the 29th Annual International Conference of the IEEE Engineering in Medicine and Biology Society*, (Lyon: IEEE), 904–907. 10.1109/IEMBS.2007.4352437 18002103

[B57] Wilson R. F. Christensen B. V. Olivari M. T. Simon A. White C. W. Laxson D. D. (1991). Evidence for structural sympathetic reinnervation after orthotopic cardiac transplantation in humans. *Circulation* 83 1210–1220. 10.1161/01.CIR.83.4.1210 2013143

[B58] Wilson R. F. Johnson T. H. Haidet G. C. Kubo S. H. Mianuelli M. (2000). Sympathetic reinnervation of the sinus node and exercise hemodynamics after cardiac transplantation. *Circulation* 101 2727–2733. 10.1161/01.CIR.101.23.2727 10851211

[B59] Wilson R. F. Laxson D. D. Christensen B. V. McGinn A. L. Kubo S. H. (1993). Regional differences in sympathetic reinnervation after human orthotopic cardiac transplantation. *Circulation* 88 165–171. 10.1161/01.cir.88.1.165 8319329

[B60] Zhang H. Holden A. V. Noble D. Boyett M. R. (2002). Analysis of the chronotropic effect of acetylcholine on sinoatrial node cells. *J. Cardiovasc. Electrophysiol.* 13 465–474. 10.1046/j.1540-8167.2002.00465.x 12030529

