# Supplementary material for: Changes in Resting and Exercise Hemodynamics Early After Heart Transplantation: A Simulation Perspective
Source: Front Physiol. 2020 Nov 6;11:579449. doi: 10.3389/fphys.2020.579449 (PMC7677526; doi:10.3389/fphys.2020.579449)
Supplement: Supplementary file 1 [file Data_Sheet_1.PDF]

Supplementary Material

1 Medication Regimen

**Immunosuppressants**	
Cyclosporine	Doering et al., 1996; Hayman et al., 2010; Notarius et al., 1998; Smith et al., 1990; Bernardi et al., 1989; Pflugfelder et al., 1987; Andreassen et al., 1998; Pflugfelder et al., 1988; Scott et al., 1995; Tamburino et al., 1989; Nytrøen et al., 2011; Hosenpud et al., 1989
Prednisone	Rudas et al., 1993; Geny et al., 1996; Kao et al., 1994; Nygaard et al. 2019; Kavanagh et al., 1988; Marzo et al., 1992; Notarius et al., 1998; van De Borne et al., 2001; Bernardi et al., 1989; Pflugfelder et al., 1987; Andreassen et al., 1998; Pflugfelder et al., 1988; Scott et al., 1995; Tamburino et al., 1989; Nytrøen et al., 2011; Hosenpud et al., 1989
Azathioprine	Hayman et al., 2010; Rudas et al., 1993; Geny et al., 1996; Kao et al., 1994; Kavanagh et al., 1988; Marzo et al., 1992; Notarius et al., 1998; Smith et al., 1990; Bernardi et al., 1989; van De Borne et al., 2001; Bernardi et al., 1990; Peled et al., 2017; Andreassen et al., 1998; Scott et al., 1995; Nytrøen et al., 2011; Hosenpud et al., 1989
Corticosteroids	Nygaard et al., 2019; Kavanagh et al., 1988; Labovitz et al., 1989
Tacrolimus	Hayman et al., 2010; Nygaard et al., 2019; van De Borne et al., 2001; Peled et al., 2017; Nytrøen et al., 2011
Mycophenolic acid	Hayman et al., 2010; Nygaard et al., 2019; Peled et al., 2017; Nytrøen et al., 2011
Polyclonal antibodies	Labovitz et al., 1989
**Hypertensive drugs**	
Calcium channel blockers	Doering et al., 1996; Rudas et al., 1993; Geny et al., 1996; Kao et al., 1994; Kavanagh et al., 1988; Notarius et al., 1998; van De Borne et al., 2001; Peled et al., 2017; Andreassen et al., 1998; Tamburino et al., 1989; Nytrøen et al., 2011; Hosenpud et al., 1989
ACE inhibitor	Doering et al., 1996; Rudas et al., 1993; Geny et al., 1996; Kao et al., 1994; Marzo et al., 1992; van De Borne et al., 2001; Bernardi et al., 1990; Peled et al., 2017; Andreassen et al., 1998; Nytrøen et al., 2011
Diuretics	Doering et al., 1996; Rudas et al., 1993; Geny et al., 1996; Kao et al., 1994; Kavanagh et al., 1988; Marzo et al., 1992; van De Borne et al., 2001; Peled et al., 2017;

	Pflugfelder et al., 1987; Andreassen et al., 1998; Pflugfelder et al., 1988; Hosenpud et al., 1989
Direct vasodilators	Kao et al., 1994; Kavanagh et al., 1988; Pflugfelder et al., 1987; Pflugfelder et al., 1988
Alpha-agonists	Kao et al., 1994; Hosenpud et al., 1989
Nitrates	Geny et al., 1996
Hydralazine	Bernardi et al., 1989; Peled et al., 2017
**Chronotropic medication**	
Beta blockers	Hayman et al., 2010; Notarius et al., 1998; van De Borne et al., 2001; Nytrøen et al., 2011; Hosenpud et al., 1989
**Other medication**	
Antiglycemic agents	Doering et al., 1996; Kao et al., 1994; Bernardi et al., 1989
Statins	Nygaard et al., 2019; Nytrøen et al., 2011
Digoxin	Marzo et al., 1992
Ranitidine	Bernardi et al., 1990
Furosemide	Bernardi et al., 1990
Indobufen	Bernardi et al., 1990
Adrenocorticotropic hormone	Bernardi et al., 1989
Furosemide	Bernardi et al., 1989; Tamburino et al., 1989; Nytrøen et al., 2011
Amiodaroneb	Nytrøen et al., 2011

Supplementary Table S1. Breakdown of prescribed medication per study

2 Model Equations

The model equations governing the cardiovascular system (Equations S1-S26), the autonomic cardiovascular control (Equations S27-S56) and the autonomic heart rate control (Equations S57-S175) are described here. An explanation of the parameters and their assigned values to simulate the resting and exercise hemodynamics of an early heart transplant recipient cohort and an age-gender matched healthy control group may be found in the Supplementary Tables S2-S8**.**

2.1 Heart

The cardiovascular system is modeled according to Moscato et al., 2013 and Moscato et al., 2010. The cardiac chambers are modeled as nonlinear time-varying elastances and internal resistance. Equation S1 describes the pressure-volume relationship in the atria and ventricles, whereby P represents the pressure, V the volume, φ the time-varying elastance, and R_i the internal resistance. The instantaneous ventricular elastance E(t) is given by the slope of the function φ[V(t),t], described in Equation S2, where F_{iso} represents the ventricular contraction function, while φ_a and φ_p are the active and passive pressure-volume relationships respectively. The ventricular contraction function F_{iso} is described by three cubic functions for the time intervals between 0 and X_{rise}, X_{rise} and T_{rise}, and T_{rise} and T_{sys} (Equations S3-S13).

$$P(t) = \varphi[V(t),t] - R_i \cdot \frac{dV(t)}{dt} \tag{S1}$$

$$\varphi[V(t),t] = \varphi_p[V(t)] + \varphi_a[V(t)] \cdot F_{iso}(t) \tag{S2}$$

$$T_{sys} = 1 - \left(\frac{1}{2} \cdot \left(e^{-0.01207 \cdot (HR - 40)} + e^{-0.038 \cdot (HR - 40)}\right)\right) \tag{S3}$$

$$F_{iso}(t) = \begin{cases} a_{13} \cdot t^3 + a_{12} \cdot t^2, & 0 < t < X_{rise} \\ a_{23} \cdot t^3 + a_{22} \cdot t^2 + a_{21} \cdot t + a_{20}, & X_{rise} < t < T_{rise} \\ a_{33} \cdot t^3 + a_{32} \cdot t^2 + a_{31} \cdot t + a_{30}, & T_{rise} < t < T_{sys} \\ 0, & T_{sys} < t < T \end{cases} \tag{S4}$$

$$a_{13} = \frac{-2 \cdot Y_{rise} + X_{rise} \cdot slope}{X_{rise}{}^3} \tag{S5}$$

$$a_{12} = \frac{-3 \cdot Y_{rise} + X_{rise} \cdot slope}{X_{rise}e^2} \tag{S6}$$

$$a_{23} = \frac{-2 \cdot Y_{rise} + X_{rise} \cdot slope + 2 - T_{rise} \cdot slope}{X_{rise}{}^3 + 3 \cdot X_{rise} \cdot T_{rise}{}^2 - T_{rise}{}^3 - 3 \cdot T_{rise} \cdot X_{rise}{}^2} \tag{S7}$$

$$a_{22} = \frac{s \cdot X_{rise}{}^2 - 3 \cdot X_{rise} \cdot Y_{rise} + 3 \cdot X_{rise} + X_{rise} \cdot T_{rise} \cdot s - 2 \cdot T_{rise}{}^2 \cdot s - 3 \cdot T_{rise} \cdot Y_{rise} + 3 \cdot T_{rise}}{X_{rise}e^3 + 3 \cdot X_{rise} \cdot T_{rise}{}^2 - T_{rise}{}^3 - 3 \cdot T_{rise} \cdot X_{rise}{}^2} \tag{S8}$$

$$a_{21} = \frac{T_{rise} \cdot (-X_{rise} \cdot T_{rise} \cdot s + T_{rise}{}^2 \cdot s + 2 \cdot s \cdot X_{rise}{}^2 - 6 \cdot X_{rise} \cdot Y_{rise} + 6 \cdot X_{rise})}{X_{rise}e^3 + 3 \cdot X_{rise} \cdot T_{rise}{}^2 - T_{rise}{}^3 - 3 \cdot T_{rise} \cdot X_{rise}{}^2} \tag{S9}$$

$$a_{33} = \frac{2}{-T_{rise}e^3 - 3 \cdot T_{rise} \cdot T_{sys}{}^2 + T_{sys}{}^3 + 3 \cdot T_{sys} \cdot T_{rise}{}^2} \tag{S10}$$

$$a_{32} = \frac{3 \cdot (T_{sys} + T_{rise})}{-T_{rise}e^3 - 3 \cdot T_{rise} \cdot T_{sys}{}^2 + T_{sys}{}^3 + 3 \cdot T_{sys} \cdot T_{rise}{}^2} \tag{S11}$$

$$a_{31} = \frac{6 \cdot T_{sys} \cdot T_{rise}}{-T_{rise}e^3 - 3 \cdot T_{rise} \cdot T_{sys}{}^2 + T_{sys}{}^3 + 3 \cdot T_{sys} \cdot T_{rise}{}^2} \tag{S12}$$

$$a_{30} = \frac{T_{sys}{}^2 \cdot (T_{sys} - 3 \cdot T_{rise})}{-T_{rise}{}^3 - 3 \cdot T_{rise} \cdot T_{sys}{}^2 + T_{sys}{}^3 + 3 \cdot T_{sys} \cdot T_{rise}{}^2} \tag{S13}$$

2.1.1 Left Ventricle

The function φ_a is modeled as a parabolic relationship given by Equations S14, with vortex at coordinates (V*, P*) and passing through the point (V_d, 0), where V_d is the so-called dead volume. As described in Equation S15, the function φ_p is modeled as an exponential function for volumes greater than and by a cubic function for volumes less than V_0, where c0, c1, c2, c3 are constants characterizing the cubic function, while α, β, and P_{offset} are constants describing the exponential function.

$$\varphi_a(V) = \left(1 - \frac{V^* - V^2}{V^* - V_d}\right) \cdot P^*(t) \quad \text{(S14)}$$

$$\varphi_p(V) = \begin{cases} \left(c_3 \cdot V^3 + c_2 \cdot V^2 + c_1 \cdot V + c_0\right) + P_{offset} & V_d < V < V_0 \\ \alpha \cdot V^\beta + P_{offset} & V > V_0 \end{cases} \quad \text{(S15)}$$

Parameter	Value	Unit	Description
T_{rise}	$0.6666 \cdot T_{sys}$	s	Time at which seconds phase of systolic contraction ends
X_{rise}	$0.32 \cdot T_{rise}$	s	Time at which first phase of systole contraction ends
Y_{rise}	0.42	-	$F_{iso}(t)$ for $t=X_{rise}$
slope	$1/T_{rise}$	s^{-1}	Slope of $F_{iso}(t)$ at point (X_{rise}, Y_{rise})
V*	175	mL	Volume coordinate of parabolic fnction vortex (active pressure-volume relationship φ_a)
P*	270	mmHg	Pressure coordinate of parabolic function vortex (active pressure-volume relationship φ_a)
LVM	175	gr	Left ventricular mass
V_0	$15.8 \cdot LVM/100$	mL	Ventricular volume at zero transmural pressure
P_{offset}	2.5	mmHg	Offset pressure
V_d	$4.3 \cdot LVM/100$	mL	Ventricular dead volume
P_d	-9.5	mmHg	Pressure at ventricular dead volume
c_3	-0.0011	-	Cubic function constant (passive pressure volume-relationship φ_p)
c_2	0.0454	-	Cubic function constant (passive pressure volume relationship φ_p)
c_1	0.0025	-	Cubic function constant (passive pressure volume relationship φ_p)

c_0	-11.6244	-	Cubic function constant (passive pressure volume relationship φ_p)
α	$4.127 \cdot 10^{-12}$	-	Exponential function constant (passive pressure volume relationship φ_p)
β	5.4122	-	Exponential function constant (passive pressure volume relationship φ_p)
$R_i(t)$	$3.75 \cdot 10^{-4} \cdot P_{lv}(t)$	mmHg*s /mL	Ventricular internal resistance

Supplementary Table S2. Model parameter values used for the simulation of the left ventricle. The values used are the same for both the simulation of HTxR and healthy controls.

2.1.2 Right Ventricle

Despite the different parametrization, the right ventricle is modeled as the left ventricle according to Equations S1-S4. However, the passive pressure-volume relationship φ_p is realized using a hyperbole given by Equation S5, where HR represents the heart rate, P_{ec} the extracardiac pressure, while K_r, a, and b are the contractile state and hyperbole constants respectively.

$$\varphi_p(V) = \frac{HR \cdot (P_{ec} + b) \cdot V(t) - \left(100 \cdot K_r \cdot (P_{ec} - V) + 1000 \cdot \alpha + V \cdot HR \cdot (P_{ec} + b)\right)}{HR \cdot V - (1000 \cdot K_r + V \cdot HR)} \quad (S16)$$

**Parameter**	**Value**	**Unit**	**Description**
V*	200	mL	Volume coordinate of the parabolic function vortex (active pressure-volume relationship φ_a)
P*	80	mmHg	Pressure coordinate of the parabolic function vortex (active pressure-volume relationship φ_a)
V_0	50	mL	Ventricular volume at zero transmural pressure
P_0	0	mmHg	External ventricular pressure
P_{ec}	-4	mmHg	Extracardiac pressure
K_r	14	-	Contractile state
a	5.3	-	Hyperbolic function constant (passive pressure-volume relationship φ_p)
b	3.3	-	Hyperbolic function constant (passive pressure-volume relationship φ_p)
$R_i(t)$	$1.4 \cdot 10^{-3} \cdot P_{rv}(t)$	mmHg*s/mL	Ventricular internal resistance

Supplementary Table S3. Model parameter values used for the simulation of the right ventricle. The values used are the same for both the simulation of HTxR and healthy controls.

2.1.3 Left and Right Atrium

Except for the different parameterization and that the resistance R_i is not present, the model of the atria is basically the same as that of the left ventricle given by Equations S1-S16. However, the atrial contraction leads the ventricular contraction so that F_{iso} is anticipated by a duration that corresponds to 20% of the heart period. Ultimately, the passive pressure-volume relationship is modeled as a linear relationship passing through the point (V_0, P_0) given by Equations S17 where E_{min} is the linear slope.

$$\varphi_a(V) = (V - V_0) \cdot E_{min} + P_0 \tag{S17}$$

Parameter	Value<br>Left Atrium	Value<br>Right Atrium	Unit	Description
$lead_{F_{iso}}$	20	20	% of heart period	Percentage of heart period that atrial excitation leads that of the ventricles
V*	200	200	mL	Volume coordinate of the parabolic function vortex (active pressure-volume relationship φ_a)
P*	30	10	mmHg	Pressure coordinate of the parabolic function vortex (active pressure-volume relationship φ_a)
V_0	0	0	mL	Ventricular volume at zero transmural pressure
P_0	0	-5	mmHg	External atrial pressure
E_{min}	0.125	0.100	mmHg/mL	Linear slope (passive pressure-volume relationship φ_p)

Supplementary Table S4. Model parameter values used for the simulation of the atria. The values used are the same for both the simulation of HTxR and healthy controls.

2.1.4 Heart Valves

The heart valves were modeled as direction-dependent resistances, where, in forward direction the resistance R_{dir} is low, representing valve opening, and in opposite direction R_{inv}, the resistance is very high representing valve closure. An additional inertance term L_v was added in series to the resistance. The differential equations describing the heart valves are given by equations S18 and S19.

$$P_i - P_o = R_v \cdot Q + L_v \cdot \frac{dQ}{dt} \tag{S18}$$

$$R_v = \begin{cases} R_{dir} & Q > 0 \\ R_{inv} & \text{else} \end{cases} \tag{S19}$$

Parameter	Mitral	Aortic	Tricuspid	Pulmonary	Unit	Description
R_{dir}	0.005	0.020	0.003	0.003	mmHg*s/mL	Forward direction resistance
R_{inv}	50	50	50	50	mmHg*s/mL	Reverse direction resistance
Lv	$3.15 \cdot 10^{-4}$	$5.16 \cdot 10^{-4}$	$3.15 \cdot 10^{-4}$	$5.16 \cdot 10^{-4}$	mmHg*s²/mL	Inertance

Supplementary Table S5. Model parameter values used for the simulation of the heart valves. The values used are the same for both the simulation of HTxR and healthy controls.

2.2 Systemic and Pulmonary Circulation

The systemic and pulmonary arterial loads are realized as a five-component model given by Equations S20-S23, where, whether we are looking at the systemic or pulmonary circulation, Q_{ov} is the outflow of the right or left ventricle, P_a the pulmonary artery or aortic pressure, P_v the systemic or pulmonary venous pressure, P_{it} the intrathoracic pressure, Rc, Lc and Cc the resistance, inertance, and compliance of the aorta or pulmonary artery, while Ra and Ca are the resistance and compliance of the systemic or pulmonary arterial system. The systemic and pulmonary venous returns are modeled according to Equation S14-S26, where EVs and V_{usv} are the blood extra-volume and the rest-volume respectively, while Rvs and Cvs are the resistance and compliance of the systemic or pulmonary venous returns respectively and V is the total volume in the venous vessels.

$$Q_{ov} - Q_{Lc} = Cc \cdot \frac{d \cdot (P_a - R_{Rc} \cdot Q_{RCc})}{dt} \quad \text{(S20)}$$

$$P_a - P_{a1} = Lc \cdot \frac{dQ_{Lc}}{dt} \quad \text{(S21)}$$

$$Q_{Lc} - Q_{Ra} = Ca \cdot \frac{dP_{a1}}{dt} \quad \text{(S22)}$$

$$P_{a1} - P_v = Ra \cdot Q_{Ra} \quad \text{(S23)}$$

$$\int (Q_{Ras} - Q_{Rvs})\, dt = EVs = V - V_{usv} \quad \text{(S24)}$$

$$P_{vs} = \frac{V - V_{usv}}{Cvs} \quad \text{(S25)}$$

$$P_{vs} - P_{RA} = Rv \cdot Q_{Rvs} \quad \text{(S26)}$$

Parameter	Value Systemic	Value Pulmonary	Unit	Description
Rc	0.0333	0.015	mmHg*s/mL	Aortic- or pulmonary artery resistance
Cc	1.445	3.0	mL/mmHg	Aortic- or pulmonary artery compliance
Lc	0.0093	0.0027	mmHg*s²/mL	Aortic- or pulmonary artery inertance
Ca	0.155	0.7	mL/mmHg	Systemic- or pulmonary arterial system compliance
Ra	1.066	0.7	mmHg*s/mL	Systemic- or pulmonary arterial system resistance
Rv	0.164/Pvs	0.01	mmHg*s/mL	Systemic- or pulmonary venous return resistance
Cv	50	10	mL/mmHg	Systemic- or pulmonary venous return compliance
V_{usv}	2200	200	mL	Systemic- or pulmonary venous return unstressed volume

Supplementary Table S6. Model parameter values used for the simulation of systemic and pulmonary circulation. The values used are the same for both the simulation of HTxR and healthy controls.

2.3 Autonomic Cardiovascular Control

The autonomic control, governed by Equations S27-S55, is based on Ursino and Magosso, 2003 as well as Magosso and Ursino, 2002.

2.3.1 Heart Rate Control

The heart rate is determined by the depolarization rate of the human sinoatrial node single-cell model that depends on acetylcholine (ACh) and isoprenaline (Iso) concentrations which are calculated according to Equations S27-S32.

$$\mathrm{ACh}(t) = x_v(t - D_v) \cdot c_v + \gamma_v \cdot c_{cc,v_T} + \mathrm{ACh}_{bg} \tag{S27}$$

$$\frac{dx_v}{dt} = \frac{1 - x_v}{\tau_{v_T}} \tag{S28}$$

$$x_v(t) = \left(P_{ao} - P_{ao,ref}\right) \cdot G_{a,T_v} - \left(V_{ln} - V_{ln,ref}\right) \cdot G_{p,T_v} \tag{S29}$$

$$\mathrm{Iso}(t) = x_s(t - D_s) \cdot c_s + \gamma_{sh} \cdot c_{cc,s_T} + k_{circ} \cdot c_{circ,T} + \mathrm{Iso}_{bg} \tag{S30}$$

$$\frac{dx_s}{dt} = \frac{1 - x_s}{\tau_{s_T}} \qquad \text{(S31)}$$

$$x_s(t) = \left(P_{ao} - P_{ao,ref}\right) \cdot G_{a,T_s} + \left(V_{ln} - V_{ln,ref}\right) \cdot G_{p,T_s} \qquad \text{(S32)}$$

2.3.2 Total Peripheral Resistance Control

Reflex control of total peripheral resistance was modeled according to Ursino and Magosso, 2003 as first-order dynamics (Equations S33-S37). To account for the vasodilatory effect of the metaboreflex, an additive term, that leads to an exercise intensity-dependent reduction of total peripheral resistance, was introduced to Equation S33.

$$R_{as}(t) = x_{R_{as}}(t - D_v) + x_{R_{as},cc} \qquad \text{(S33)}$$

$$\frac{dx_{R_{as}}}{dt} = \frac{1 - x_{R_{as}}}{\tau_{R_{as}}} \qquad \text{(S34)}$$

$$x_{R_{as}}(t) = \left(P_{ao} - P_{ao,ref}\right) \cdot G_{a,R_{as}} + \left(V_{ln} - V_{ln,ref}\right) \cdot G_{p,R_{as}} \qquad \text{(S35)}$$

$$\frac{dx_{R_{as},cc}}{dt} = \frac{1 - x_{R_{as},cc}}{\tau_{R_{as}}} \qquad \text{(S36)}$$

$$x_{R_{as},cc}(t) = \left(1 - I \cdot c_{cc,R_{as}}\right) \cdot R_{as,0} \qquad \text{(S37)}$$

2.3.3 Venous Unstressed Volume Control

Control of venous unstressed volume was modeled as described by Ursino and Magosso, 2003. The influence of central command was incorporated by assuming a linear relationship of venous unstressed volume and exercise intensity (Equations S38-S40).

$$V_{usv}(t) = x_{V_{usv}}(t - D_v) \cdot \left(1 + I \cdot c_{cc,V_{usv}}\right) + V_{usv,0} \qquad \text{(S38)}$$

$$\frac{dx_{V_{usv}}}{dt} = \frac{1 - x_{V_{usv}}}{\tau_{V_{usv}}} \qquad \text{(S39)}$$

$$x_{V_{usv}}(t) = \left(P_{ao} - P_{ao,ref}\right) \cdot G_{a,V_{usv}} \qquad \text{(S40)}$$

2.3.4 Ventricular Elastance Control

Modulation of ventricular elastance was modeled according to Ursino and Magosso, 2003. To model the influence of central command we assumed a linear relationship between ventricular elastance and exercise intensity (Equations S41-S44).

$$E_i(t) = x_{E_i}\left(t - D_{E_i}\right) \cdot \left(1 + I \cdot c_{cc,E_i}\right) \quad \text{(S41)}$$

$$\frac{dx_{E_{max,i}}}{dt} = \frac{1 - x_{E_i}}{\tau_{E_i}} \quad \text{(S42)}$$

$$x_{E_i}(t) = -\left(P_{ao} - P_{ao,ref}\right) \cdot G_{a,E_i} + E_{i,0} \quad \text{(S43)}$$

$$i = lv,rv \quad \text{(S44)}$$

2.3.5 Arterial Compliance Control

As given in Equations S45 and S46, to model the reduction of aortic and arterial compliance during exercise, we assumed a linear reciprocal relationship with exercise intensity.

$$C_{as} = \left(1 - I \cdot C_{C_{as}}\right) \cdot C_{as,0} \quad \text{(S45)}$$

$$C_{ao} = \left(1 - I \cdot C_{C_{ao}}\right) \cdot C_{ao,0} \quad \text{(S46)}$$

2.4 Circulating Catecholamines

Circulating catecholamines released by the adrenal medulla and spillover from vascular beds were modeled as a first-order dynamic system. As described by Equations S47 and S48, the release solely depends on sympathetic activity.

$$\frac{dk_{circ}}{dt} = \frac{nora - k_{circ}}{\tau_{k_{circ}}} \quad \text{(S47)}$$

$$\frac{dnora}{dt} = c_{nora}\frac{\left(P_{ao} - P_{ao,ref}\right) \cdot G_{a,am} + \left(V_{ln} - V_{ln,ref}\right) \cdot G_{p,am} + \gamma_{sh} \cdot c_{cc,am,sh} + \gamma_{sp} \cdot c_{cc,am,sp}}{\tau_{k_{circ}}} \quad \text{(S48)}$$

2.4.1 Central Command

The effect of the central command is given by the term γ_i whose dependence on exercise intensity I is described by a sigmoidal relationship (Equation S49). γ_{min} and γ_{max} define the upper and lower bound, I_0, the value of exercise intensity at the central point of the sigmoid and the constant k_{cc} is related to the slope of the central point of the sigmoidal function (Magosso and Ursino, 2002). The subscripts v,sh, and sp indicate the influence of central command on vagal activity, the heart and on vascular beds (Equation S50).

$$\gamma_i = \frac{\gamma_{i,min} + \gamma_{i,max} \cdot e^{\frac{I - I_{0,i}}{k_{cc,i}}}}{1 + e^{\frac{I - I_{0,i}}{k_{cc,i}}}} \tag{S49}$$

$$i = v,sh,sp \tag{S50}$$

2.4.2 Respiration

The temporal effects of respiration on intrathoracic and abdominal pressures were integrated into the model according to Ursino and Magosso, 2003 and are given by Equations S51-S55, where T_R is the respiratory period, T_i and T_e, the inspiration and expiration duration respectively, α represents the fraction in the respiratory cycle, and V_{ln} is the instantaneous lung volume.

$$P_{thor} = \begin{cases} -5 \cdot \alpha \cdot \frac{T_R}{T_i} - 4, & 0 < \alpha < \frac{T_R}{T_i} \\ -5 \cdot \frac{T_i + T_e - \alpha \cdot T_R}{T_e} - 4, & \frac{T_R}{T_i} < \alpha < \frac{T_i + T_e}{T_R} \\ -4, & \frac{T_i + T_e}{T_R} < \alpha < \frac{T_i + T_e}{T_R} \end{cases} \tag{S51}$$

$$P_{abd} = \begin{cases} -2.5 \cdot \alpha \cdot \frac{T_R}{T_i/2} - 4, & 0 < \alpha < \frac{T_i/2}{T_R} \\ -2.5, & \frac{T_i/2}{T_R} < \alpha < \frac{T_i}{T_R} \\ -2.5 \cdot \frac{T_i + T_e - \alpha \cdot T_R}{T_e} - 4, & \frac{T_i}{T_R} < \alpha < \frac{T_i + T_e}{T_R} \\ 0, & \frac{T_i + T_e}{T_R} < \alpha < 1 \end{cases} \tag{S52}$$

$$\frac{d\varepsilon}{dt} = \frac{1}{T_{resp}} \tag{S53}$$

$$\alpha(t) = \mathrm{frac}(\varepsilon) \tag{S54}$$

$$V_{ln} = 1.9 - 0.1 \cdot P_{thor} \tag{S55}$$

2.4.3 Respiratory Exercise Response

The respiratory exercise response reflected by the change in respiration period T_R was modeled by a gradual reduction of the baseline respiration period $T_{R,max}$, to finally reach the peak respiration interval $T_{R,min}$ at 100% exercise intensity (Equation S56).

$$T_R = \left(T_{R,max} - T_{R,min}\right) \cdot I + T_{R,max} \qquad \text{(S56)}$$

2.4.4 Model Parameter Values

Parameter	Value Healthy	Value HTxR	Unit	Description
$P_{ao,ref}$	91	91	mmHg	Arterial baroreflex setpoint
$V_{ln,ref}$	2.1	2.1	L	Pulmonary stretch reflex setpoint
**Heart rate control**				
G_{aTv}	0.015	0	$mmHg^{-1}$	Vagal arterial baroreflex gain
G_{pTv}	0.54	0	L^{-1}	Vagal pulmonary stretch reflex gain
G_{aTs}	0.016	0.0007	$mmHg^{-1}$	Sympathetic arterial baroreflex reflex gain
G_{pTs}	0	0	L^{-1}	Sympathetic pulmonary stretch reflex gain
D_{Tv}	0.5	0.5	s	Delay of vagal effect
D_{Ts}	3.0	3.0	s	Delay of sympathetic effect
τ_V	0.8	0.8	s	Time constant of vagal effect
τ_S	1.8	1.8	s	Time constant of sympathetic effect
$c_{cc,v,T}$	$9.2 \cdot 10^{-6}$	0	1	Gain for effect of central comm. on vagal activity
$c_{cc,s,T}$	$5 \cdot 10^{-8}$	0	1	Gain for effect of central comm. on symp. activity
c_V	$2.6 \cdot 10^{-5}$	$2.6 \cdot 10^{-5}$	1	Gain to correlate vagal outflow to [ACh]
c_S	$1 \cdot 10^{-9}$	$1 \cdot 10^{-9}$	1	Gain to correlate sympathetic outflow to [Iso]
ACh_{bg}	30.8	30.8	nM	Baseline concentration of acetylcholine
Iso_{bg}	3.5	3.5	nM	Baseline concentration of isoprenaline
**Total peripheral resistance control**				
G_{aRas}	0.16	0.16	$mmHg^{-1}$	Sympathetic arterial baroreflex reflex gain
G_{pRas}	0.04	0.04	L^{-1}	Sympathetic pulmonary stretch reflex gain
$R_{as,0}$	0.97	1.12	mmHg*s/mL	Baseline total peripheral resistance
$c_{cc,Ras}$	0.12	0.12	1	Central command gain
D_{Ras}	2	2	s	Delay of sympathetic effect
τ_{Ras}	5.1	5.1	s	Time constant of sympathetic effect
**Venous unstressed volume control**				
G_{aVusv}	10.6	10.6	$mmHg^{-1}$	Sympathetic arterial baroreflex reflex gain
$c_{cc,Vusv}$	1.2	1.2	1	Sympathetic pulmonary stretch reflex gain
D_{Vusv}	1.2	1.2	s	Delay of sympathetic effect
τ_{Vusv}	2.0	2.0	s	Time constant of sympathetic effect
$V_{usv,0}$	2200	2200	mL	Baseline venous unstressed volume

**Ventricular elastance control (lv, left ventricle, rv, right ventricle)**				
G_{aElv}	3	0.15	$mmHg^{-1}$	Sympathetic arterial baroreflex reflex gain
G_{aErv}	3	0.15	$mmHg^{-1}$	Sympathetic arterial baroreflex reflex gain
$c_{ccE,lv}$	30	30	1	Central command gain
$c_{ccE,rv}$	30	30	1	Central command gain
$D_{El,v}$	2	2	s	Delay of sympathetic effect
$D_{e.rv}$	2	2	s	Delay of sympathetic effect
$\tau_{E,lv}$	0.25	0.25	s	Time constant of sympathetic effect
$\tau_{E,rv}$	0.25	0.25	s	Time constant of sympathetic effect
$E_{lv,0}$	1.15	1.15	mmHg/mL	Baseline elastance
$E_{rv,0}$	1.15	1.15	mmHg/mL	Baseline elastance
**Compliance control**				
c_{Cas}	-0.96	-0.96	1	Central command gain
$C_{as,0}$	1.155	1.155	ml/mmHg	Initial arterial compliance
C_{Cao}	-0.96	-0.96	1	Central command gain
$C_{ao,0}$	1.445	1.445	mmHg/ml	Initial aortic compliance
**Circulating catecholamines**				
$G_{a,am}$	0.016	0.016	$mmHg^{-1}$	Sympathetic arterial baroreflex gain
$G_{p,am}$	0	0	L^{-1}	Sympathetic pulmonary stretch reflex gain
$C_{cc,am,sh}$	1.0	1.0	1	Central command gain (acting directly on heart)
$C_{cc,am,sp}$	1.0	1.0	1	Central command gain (acting on peripheral res.)
C_{nora}	0.2	0.2	1	Proportional gain of noradrenaline release
τ_{kcirc}	1.32	1.32	s	Time constant for release of circulating catechol.
τ_{nora}	6	6	s	Time constant of noradrenaline release
**Central command**				
$\gamma_{v,min}$	-0.0008	-0.0008	1	Lower bound of sigmoidal function
$\gamma_{v,max}$	3.04	3.04	1	Upper bound of sigmoidal function
$\gamma_{sh,min}$	-0.0283	-0.283	1	Lower bound of sigmoidal function
$\gamma_{sh,max}$	9	9	1	Upper bound of sigmoidal function
$\gamma_{sp,min}$	-0.037	-0.037	1	Lower bound of sigmoidal function
$\gamma_{sp,max}$	5.5	5.5	1	Upper bound of sigmoidal function
$I_{0,v}$	0.126	0.126	1	Intensity at the central point of the sigmoidal function.
$I_{0,sh}$	0.658	0.658	1	Intensity at the central point of the sigmoidal function

$I_{0,sp}$	0.65	0.65	1	Intensity at the central point of the sigmoidal function.
$k_{cc,v}$	0.0648	0.0648	1	Slope of the central point of the sigmoidal function.
$k_{cc,h}$	0.114	0.114	1	Slope of the central point of the sigmoidal function.
$k_{cc,sp}$	0.13	0.13	1	Slope of the central point of the sigmoidal function.
**Respiration**				
$T_{R,max}$	5	5	s	Maximum respiration period (rest)
$T_{R,min}$	0.8333	0.8333	s	Minimum respiration period (peak exercise)
T_i	$0.5 \cdot T_R$	$0.5 \cdot T_R$	s	Inspiration duration
T_e	$0.5 \cdot T_R$	$0.5 \cdot T_R$	s	Expiration duration

Supplementary Table S7. Model parameter values used for the simulation of healthy and heart transplanted individuals.

2.5 Intrinsic Heart Rate Control

The intrinsic heart rate control is realized as a single-cell human sinoatrial (SA) node model that is governed by Equations S57-S175. The model is based on the work of Fabbri et al., 2017; Li et al., 2014 and Zhang et al., 2002. The heart rate is calculated as the reciprocal of the time differences of consecutive SA nod action potentials.

2.5.1 Membrane Potential

The transmembrane potential of the sinoatrial node is given by Equation S57 and determined by the membrane capacitance C and the sum of transmembrane currents. The equations describing the transmembrane currents are given in Equations S58-S175, while parameter descriptions and their values used for simulation can be found in Table S8.

$$\frac{dV}{dt} = -\frac{1}{C} \cdot \left(I_f + I_{CaL} + I_{CaT} + I_{Kr} + I_{Ks} + I_{K,Ach} + I_{to} + I_{Na} + I_{NaK} + I_{NaCa} + I_{Kur}\right) \quad \text{(S57)}$$

2.5.2 Hyperpolarization-activated Current (I_f)

The hyperpolarization-activated “funny” current I_f is target of sympathetic and parasympathetic modulation.

$$I_f = I_{fNa} + I_{fK} \quad \text{(S58)}$$

$$I_{fNa} = y \cdot g_{f_{Na}} \cdot (V - E_{Na}) \quad \text{(S59)}$$

$$I_{fK} = y \cdot g_{f_K} \cdot (V - E_K) \quad \text{(S60)}$$

$$S_{f'} = S_{f',max} \cdot \frac{[Iso]^{n_{f'}}}{K_{0.5,f'}^{\,n_{f'}} + [Iso]^{n_{f'}}} \quad \text{(S61)}$$

$$S_f = S_{f,max} \cdot \frac{[ACh]^{n_f}}{{K_{0.5,f}}^{n_f} + [ACh]^{n_f}} \tag{S62}$$

$$y_\infty = \begin{cases} 0.01329 + \dfrac{0.99921}{1 + e^{\frac{V + 97.134}{8.1752}}} & V < -80 - S_f - S_{f'} \\ 0.0002501 \cdot e^{\frac{-V}{12.861}} & \text{else} \end{cases} \tag{S63}$$

$$\tau_y = \frac{1}{\dfrac{0.36 \cdot (V + 148.8 + S_f - S_{f'})}{e^{0.066 \cdot (V + 148.8 + S_f - S_{f'})} - 1} + \dfrac{0.1 \cdot (V + 87.3 + S_f - S_{f'})}{1 - e^{-0.2 \cdot (V + 87.3 + S_f - S_{f'})}}} \tag{S64}$$

$$\frac{dy}{dt} = \frac{y_\infty - y}{\tau_y} \tag{S65}$$

2.5.3 L-type Ca^{2+} Current (I_{CaL})

The L-type calcium current I_{CaL} is target of sympathetic and parasympathetic modulation.

$$I_{CaL} = (I_{siCa} + I_{siK} + I_{siNa}) \cdot \left(1 - \frac{0.31 \cdot [ACh]}{K_{0.5,Ca} + [ACh]}\right) \cdot \left(1 + \frac{[Iso]}{K_{0.5,Ca'} + [Iso]}\right) \tag{S66}$$

$$I_{siCa} = \frac{2 \cdot P_{CaL} \cdot V}{RTonF \cdot (1 - e^{\frac{-2 \cdot V}{RTonF}})} \cdot \left(Ca_{sub} - Cao \cdot e^{\frac{-2*V}{RTonF}}\right) \cdot dL \cdot fL \cdot fCa \tag{S67}$$

$$I_{siK} = \frac{0.000365 \cdot P_{CaL} \cdot V}{RTonF \cdot (1 - e^{\frac{-V}{RTonF}})} \cdot \left(Ki - Ko \cdot e^{\frac{*V}{RTonF}}\right) \cdot dL \cdot fL \cdot fCa \tag{S68}$$

$$I_{siNa} = \frac{0.0000185 \cdot P_{CaL} \cdot V}{RTonF \cdot (1 - e^{\frac{-V}{RTonF}})} \cdot \left(Nai - Nao \cdot e^{\frac{-V}{RTonF}}\right) \cdot dL \cdot fL \cdot fCa \tag{S69}$$

$$S_{Ca'} = S_{Ca',max} \cdot \frac{[Iso]^{n_{Ca'}}}{{K_{0.5,Ca'}}^{n_{Ca'}} + [Iso]^{n_{Ca'}}} \tag{S70}$$

$$dL_\infty = \frac{1}{1 + \exp\left(\dfrac{-\left(V + 16.45 + s_{Ca'}\right)}{4.337 \cdot \left(\dfrac{1 + 2.4 \cdot s_{Ca'}}{100}\right)}\right)} \tag{S71}$$

$$\alpha_{dL} = \frac{-0.02839 \cdot (V + 41.8)}{e^{\frac{-(V + 41.8)}{2.5}} - 1} - \frac{-0.0849 \cdot (V + 6.8)}{e^{\frac{-(V + 6.8)}{4.8}} - 1} \tag{S72}$$

$$\beta_{dL} = \frac{0.01143 \cdot (V + 1.8)}{e^{\frac{V + 1.8}{2.5}} - 1} \tag{S73}$$

$$\tau_{dL} = \frac{0.001}{\alpha_{dL} + \beta_{dL}} \tag{S74}$$

$$\frac{ddL}{dt} = \frac{dL_{\infty} - dL}{\tau_{dL}} \tag{S75}$$

$$f_{Ca_{\infty}} = \frac{Km_{fCa}}{Km_{fCa} + Ca_{sub}} \tag{S76}$$

$$\tau_{fCa} = \frac{0.001 \cdot f \cdot fCa_{\infty}}{\alpha_{fCa}} \tag{S77}$$

$$\frac{dfCa}{dt} = \frac{dfCa_{\infty} - dfCa}{\tau_{f_{Ca}}} \tag{S78}$$

$$fL_{\infty} = \frac{1}{1 + e^{\frac{V + 37.4}{5.3}}} \tag{S79}$$

$$\tau_{fL} = 0.001 \cdot \left(44.3 + 230 \cdot e^{-\left(\frac{V + 36}{10}\right)^2}\right) \tag{S80}$$

$$\frac{dfL}{dt} = \frac{fL_{\infty} - fL}{\tau_{fL}} \tag{S81}$$

2.5.4 T-type Ca^{2+} Current (I_{CaT})

$$I_{CaT} = \frac{2 \cdot P_{CaT} \cdot V}{RTonF \cdot \left(1 - e^{\frac{-2*V}{RTonF}}\right)} \cdot \left(Ca_{sub} - Cao \cdot e^{\frac{-2*V}{RTonF}}\right) \cdot dT \cdot fT \tag{S82}$$

$$dT_{\infty} = \frac{1}{1 + e^{\frac{-(V + 38.3)}{5.5}}} \tag{S83}$$

$$\tau_{dT} = \frac{0.001}{1.068 \cdot e^{\frac{V + 38.3}{30}} + 1.068 \cdot e^{\frac{-(V + 38.3)}{30}}} \tag{S84}$$

$$\frac{ddT}{dt} = \frac{dT_{\infty} - dT}{\tau_{dT}} \tag{S85}$$

$$fT_{\infty} = \frac{1}{1 + e^{\frac{V + 58.7}{3.8}}} \tag{S86}$$

$$\tau_{fT} = \frac{1}{16.67 \cdot e^{\frac{-(V + 75)}{83.3}} + 16.67 \cdot e^{\frac{V + 75}{15.38}}} \tag{S87}$$

$$\frac{dfT}{dt} = \frac{fT_{\infty} - fT}{\tau_{fT}} \tag{S88}$$

2.5.5 Rapidly-activating Delayed Rectifier K+ Current (I_{Kr})

$$I_{Kr} = g_{Kr} \cdot (V - E_K) \cdot (0.9 \cdot paF + 0.1 \cdot paS) \cdot piy \qquad (S89)$$

$$paF_\infty = paS_\infty = pa_\infty = \frac{1}{1 + e^{\frac{-(V + 10.0144)}{7.6607}}} \qquad (S90)$$

$$\tau_{paS} = \frac{0.84655354}{4.2 \cdot e^{\frac{V}{17}} + 0.15 \cdot e^{\frac{-V}{21.6}}} \qquad (S91)$$

$$\tau_{paS} = \frac{1}{30 \cdot e^{\frac{V}{10}} + e^{\frac{-V}{12}}} \qquad (S92)$$

$$\frac{dpaS}{dt} = \frac{pa_\infty - paS}{\tau_{paS}} \qquad (S93)$$

$$\frac{dpaF}{dt} = \frac{pa_\infty - paF}{\tau_{paF}} \qquad (S94)$$

$$\tau_{piy} = \frac{1}{100 \cdot e^{\frac{-V}{54.645}} + 656 \cdot e^{\frac{V}{106.157}}} \qquad (S95)$$

$$\frac{dfT}{dt} = \frac{fT_\infty - fT}{\tau_{fT}} \qquad (S96)$$

$$piy_\infty = \frac{1}{1 + e^{\frac{V + 28.6}{17.1}}} \qquad (S97)$$

$$\frac{dpiy}{dt} = \frac{piy_\infty - piy}{\tau_{piy}} \qquad (S98)$$

2.5.6 Slowly-activating Delayed Rectifier K+ Current (I_{Ks})

The slowly activating delayed rectifier potassium current I_{Ks} is target of sympathetic modulation.

$$I_{Ks} = \left(1 + \frac{[Iso] \cdot f_{K_{max}}}{[Iso] + K_{0.5,g_k{}'}}\right) \cdot g_{Ks} \cdot (V - E_{Ks}) \cdot n^2 \qquad (S99)$$

$$s_{Kr'} = S_{Kr',max} \cdot \frac{[Iso]^{n_{Kr'}}}{K_{0.5,Kr'} + [Iso]^{n_{Kr'}}} \qquad (S100)$$

$$n_\infty = \sqrt{\frac{1}{1 + e^{\frac{-(V + 0.6383) + s_{Kr'}}{10.7071}}}} \tag{S101}$$

$$\tau_n = \frac{1}{\alpha_n + \beta_n} \tag{S102}$$

$$\alpha_n = \frac{28}{1 + e^{\frac{-(V - 40)}{3}}} \tag{S103}$$

$$\beta_n = e^{\frac{-(V - 5)}{25}} \tag{S104}$$

$$\frac{dn}{dt} = \frac{n_\infty - n}{\tau_n} \tag{S105}$$

2.5.7 ACh-activated K⁺ Current ($I_{K,ACh}$)

The ACh-activated potassium current $I_{K,ACh}$ is involved in the parasympathetic cardiac pathway and is activated when [ACh] is non-zero.

$$I_{K,Ach} = \begin{cases} g_{K,Ach} \cdot (V - E_K) \cdot \left(1 + e^{\frac{V + 20}{20}}\right) \cdot a & [ACh] > 0 \\ 0 \text{ else} \end{cases} \tag{S106}$$

$$\alpha_a = \frac{3.5988 - 0.025641}{1 + e^{\frac{0.0000012155}{(ACh)^{1.6951}}}} + 0.025641 \tag{S107}$$

$$\beta_a = 10 \cdot e^{0.0133 \cdot (V + 40)} \tag{S108}$$

$$\alpha_\infty = \frac{\alpha_a}{\alpha_a + \beta_a} \tag{S109}$$

$$\tau_\alpha = \frac{1}{\alpha_a + \beta_a} \tag{S110}$$

$$\frac{da}{dt} = \frac{a_\infty - a}{\tau_a} \tag{S111}$$

2.5.8 Transient Outward K⁺ Current (I_{to})

$$I_{to} = g_{to} \cdot (V - E_K) \cdot q \cdot r \tag{S112}$$

$$q_\infty = \frac{1}{1 + e^{\frac{V + 49}{13}}} \tag{S113}$$

$$\tau_\alpha = 0.001 \cdot 0.6 \cdot \left(\frac{65.17}{0.057 \cdot e^{-0.08 \cdot (V + 44)} + 0.065 \cdot e^{0.1 \cdot (V + 45.93)}} + 10.1\right) \quad (S114)$$

$$\frac{dq}{dt} = \frac{q_\infty - q}{\tau_q} \quad (S115)$$

$$r_\infty = \frac{1}{1 + e^{\frac{-(V - 19.3)}{15}}} \quad (S116)$$

$$\tau_r = 0.001 \cdot 0.66 \cdot 1.4 \cdot \left(\frac{15.59}{1.037 \cdot e^{0.09 \cdot (V + 30.61)} + 0.369 \cdot e^{-0.12 \cdot (V + 23.84)}} + 2.98\right) \quad (S117)$$

$$\frac{dr}{dt} = \frac{r_\infty - r}{\tau_r} \quad (S118)$$

2.5.9 Na^+ Current (I_{Na})

$$I_{Na} = g_{Na} \cdot m^3 \cdot h \cdot (V - E_{mh}) \quad (S119)$$

$$m_\infty = \frac{1}{1 + e^{\frac{-(V + 42.0504)}{8.3106}}} \quad (S120)$$

$$E0_m = V + 41 \quad (S121)$$

$$\alpha_m = \frac{200 \cdot E0_m}{1 - e^{-0.1 \cdot E0_m}} \quad (S122)$$

$$\beta_m = 8000 \cdot e^{-0.056 \cdot (V + 66)} \quad (S123)$$

$$\tau_m = \frac{1}{\alpha_m + \beta_m} \quad (S124)$$

$$\frac{dm}{dt} = \frac{m_\infty - m}{\tau_m} \quad (S125)$$

$$h_\infty = \frac{1}{1 + e^{\frac{V + 69.804}{4.4565}}} \quad (S126)$$

$$\alpha_h = 20 \cdot e^{-0.125 \cdot (V + 75)} \quad (S127)$$

$$\beta_h = \frac{2000}{320 \cdot e^{-0.1 \cdot (V + 75)} + 1} \quad (S128)$$

$$\tau_h = \frac{1}{\alpha_h + \beta_h} \quad (S129)$$

$$\frac{dh}{dt} = \frac{h_\infty - h}{\tau_h} \quad (S130)$$

2.5.10 Na^{2+}/K^{+} Pump Current (I_{NaK})

The sodium/potassium pump current I_{NaK} is involved in the sympathetic cardiac control and is increased by 20% if [Iso] is non-zero.

$$I_{NaK} = \begin{cases} 1.2 \cdot I_{NaK,max} \cdot \left(1 + \left(\frac{K_{m_{Kp}}}{Ko}\right)^{1.2}\right)^{-1} \cdot \left(1 + \left(\frac{K_{m_{Nap}}}{Nai}\right)^{1.3}\right)^{-1} \cdot \left(1 + e^{\frac{-(V - E_{Na} + 110)}{20}}\right)^{-1} & [Iso] > 0 \\ I_{NaK,max} \cdot \left(1 + \left(\frac{K_{m_{Kp}}}{Ko}\right)^{1.2}\right)^{-1} \cdot \left(1 + \left(\frac{K_{m_{Nap}}}{Nai}\right)^{1.3}\right)^{-1} \cdot \left(1 + e^{\frac{-(V - E_{Na} + 110)}{20}}\right)^{-1} & \text{else} \end{cases} \quad \text{(S131)}$$

2.5.11 Na^{+}/Ca^{2+} Exchanger Current (I_{NaCa})

$$I_{NaCa} = \frac{K_{NaCa} \cdot (x2 \cdot k21 - x1 \cdot k12)}{x1 + x2 + x3 + x4} \quad \text{(S132)}$$

$$x1 = k41 \cdot k34 \cdot (k23 + k21) + k21 \cdot k32 \cdot (k43 + k41) \quad \text{(S133)}$$

$$x2 = k32 \cdot k43 \cdot (k14 + k12) + k41 \cdot k12 \cdot (k34 + k32) \quad \text{(S134)}$$

$$x3 = k14 \cdot k43 \cdot (k23 + k21) + k12 \cdot k23 \cdot (k43 + k41) \quad \text{(S135)}$$

$$x4 = k23 \cdot k34 \cdot (k14 + k12) + k14 \cdot k21 \cdot (k34 + k32) \quad \text{(S136)}$$

$$k43 = \frac{Nai}{K3ni + Nai} \quad \text{(S137)}$$

$$k12 = \frac{\frac{Nai}{Kci} \cdot e^{\frac{-Qci*V}{RTonF}}}{di} \quad \text{(S138)}$$

$$k14 = \frac{\frac{\frac{Nai}{K1ni} \cdot Nai}{K2ni} \cdot \left(1 + \frac{Nai}{K3ni}\right) \cdot e^{\frac{Qn \cdot V}{2 \cdot RTonF}}}{di} \quad \text{(S139)}$$

$$k41 = e^{\frac{-Qn \cdot V}{2 \cdot RTonF}} \quad \text{(S140)}$$

$$di = 1 + \frac{Ca_{sub}}{Kci} \cdot \left(1 + e^{\frac{-Qci \cdot V}{RTonF}} + \frac{Nai}{Kcni}\right) + \frac{Nai}{K1ni} \cdot \left(1 + \frac{Nai}{K2ni} \cdot \left(1 + \frac{Nai}{K3ni}\right)\right) \quad \text{(S141)}$$

$$k34 = \frac{Nao}{K3no + Nao} \quad \text{(S142)}$$

$$k21 = \frac{\frac{Cao}{Kco} \cdot e^{\frac{Qco \cdot V}{2 \cdot RTonF}}}{do} \tag{S143}$$

$$k23 = \frac{\frac{Nao^2}{K1no \cdot K2no} \cdot \left(1 + \frac{Nao}{K3no}\right) \cdot e^{\frac{-Qn \cdot V}{2 \cdot RTonF}}}{do} \tag{S144}$$

$$k32 = e^{\frac{Qn \cdot V}{2 \cdot RTonF}} \tag{S145}$$

$$do = 1 + \frac{Cao}{Kco} * \left(1 + e^{\frac{Qco*V}{RTonF}} + \frac{Nao}{K1no}\right) + \frac{Nai}{K1ni} * \left(1 + \frac{Nao}{K2no} * \left(1 + \frac{Nao}{K3no}\right)\right) \tag{S146}$$

2.5.12 Ultra-rapid Activating Delayed Rectifier K^+ Current (I_{Kur})

$$I_{Kur} = g_{Kur} \cdot r_{Kur} \cdot s_{Kur}(V - E_K) \tag{S147}$$

$$\frac{dr_{Kur}}{dt} = \frac{r_{Kur_\infty} - r_{Kur}}{\tau_{r_{Kur}}} \tag{S148}$$

$$r_{Kur_\infty} = \frac{1}{1 + e^{\frac{V + 6}{-8.6}}} \tag{S149}$$

$$\tau_{r_{Kur}} = \frac{0.009}{1 + e^{\frac{V + 5}{12}}} + 0.005 \tag{S150}$$

$$\frac{ds_{Kur}}{dt} = \frac{s_{Kur_\infty} - s_{Kur}}{\tau_{s_{Kur}}} \tag{S151}$$

$$s_{Kur_\infty} = \frac{1}{1 + e^{\frac{V + 7.5}{10}}} \tag{S152}$$

$$\tau_{s_{Kur}} = \frac{0.59}{1 + e^{\frac{V + 60}{10}}} + 3.05 \tag{S153}$$

2.5.13 Ca^{2+} Release Flux from Sarcoplasmic Reticulum via Ryanodine Receptors

$$J_{SRCarel} = ks \cdot O \cdot (Ca_{jsr} - Ca_{sub}) \tag{S154}$$

$$kCaSR = MaxSR - \frac{MaxSR - MinSR}{1 + \left(\frac{EC50_{SR}}{Ca_{jsr}}\right)^{HSR}} \tag{S155}$$

$$koSRCa = \frac{koCa}{kCaSR} \tag{S156}$$

$$kiSRCa = kiCa \cdot kCaSR \tag{S157}$$

$$\frac{dR}{dt} = kim \cdot RI - kiSRCa \cdot Ca_{sub} \cdot R - (koSRCa \cdot Ca_{sub}^2 \cdot R - kom \cdot O) \quad \text{(S158)}$$

$$\frac{dO}{dt} = koSRCa \cdot Ca_{sub}^2 \cdot R - kom \cdot O - (kiSRCa \cdot Ca_{sub} \cdot O - kim \cdot I) \quad \text{(S159)}$$

$$\frac{dI}{dt} = kiSRCa \cdot Ca_{sub} \cdot O - kim \cdot I - (kom \cdot I - koSRCa \cdot Ca_{sub}^2 \cdot RI) \quad \text{(S160)}$$

$$\frac{dRI}{dt} = kom \cdot I - koSRCa \cdot Ca_{sub}^2 \cdot RI - (kim \cdot RI - kiSRCa \cdot Ca_{sub} \cdot R) \quad \text{(S161)}$$

2.5.14 Intracellular Ca^{2+} Fluxes

The intracellular Ca^{2+} fluxes are influence by the sympathetic and parasympathetic nervous system by modulation of the rate constant P_{up}.

$$P_{up} = 0.7 \cdot \left(\frac{[ACh]}{9 \cdot 10^{-5} + [ACh]} - \frac{[Iso]}{9 \cdot 10^{-5} + [Iso]}\right) \quad \text{(S162)}$$

$$J_{Ca_{dif}} = \frac{Ca_{sub} - Cai}{\tau_{dif_{Ca}}} \quad \text{(S163)}$$

$$J_{up} = \frac{P_{up}}{1 + e^{\frac{-(Cai - K_{up})}{slope_{up}}}} \quad \text{(S164)}$$

$$J_{tr} = \frac{Ca_{nsr} - Ca_{jsr}}{\tau_{tr}} \quad \text{(S165)}$$

2.5.15 Ca^{2+} Buffering

$$\frac{dfTC}{dt} = kf_{TC} \cdot Cai \cdot (1 - f\,TC) - kb_{TC} \cdot fTC \quad \text{(S166)}$$

$$\frac{dfTMC}{dt} = kf_{TMC} \cdot Cai \cdot \big(1 - (fTMC + fTMM)\big) - kb_{TMC} \cdot fTMC \quad \text{(S167)}$$

$$\frac{dfTMM}{dt} = kf_{TMM} \cdot Mgi \cdot (1 - (fTMC + fTMM)) - kb_{TMM} \cdot fTMM \quad \text{(S168)}$$

$$\frac{dfCMi}{dt} = kf_{CM} \cdot Cai \cdot (1 - fCMi) - kb_{CM} \cdot fCMi \quad \text{(S169)}$$

$$\frac{dfCMs}{dt} = kf_{CM} \cdot Ca_{sub} \cdot (1 - fCMs) - kb_{CM} \cdot fCMs \quad \text{(S170)}$$

$$\frac{dfCQ}{dt} = kf_{CQ} \cdot Cajsr \cdot (1 - fCQ) - kb_{CQ} \cdot fCQ \quad \text{(S171)}$$

2.5.16 Dynamics of Ca^{2+} Concentrations in Cell Compartments

$$\frac{dCai}{dt} = \frac{J_{Ca_{dif}} \cdot V_{sub} - J_{up} \cdot V_{nsr}}{V_i} - (CM_{tot} \cdot \delta_{fCMi} + TC_{tot} \cdot \delta_{fTC} + TMC_{tot} \cdot \delta_{fTMC}) \quad \text{(S172)}$$

$$\frac{dCa_{sub}}{dt} = \frac{J_{SRCarel} \cdot V_{jsr}}{V_{sub}} - \left(\frac{i_{siCa} + i_{CaT} - 2 \cdot i_{NaCa}}{2 \cdot F \cdot V_{sub}} + J_{Ca_{dif}} + CM_{tot} \cdot \delta_{fCMs}\right) \quad (S173)$$

$$\frac{dCa_{nsr}}{dt} = J_{up} - \frac{J_{tr} \cdot V_{jsr}}{V_{nsr}} \quad (S174)$$

$$\frac{dCa_{jsr}}{dt} = J_{tr} - (J_{SRCarel} + CQ_{tot} \cdot \delta_{fCQ}) \quad (S175)$$

Parameter	Value	Unit	Description
C	57	pF	Membrane capacitance
L_{cell}	67	μm	Cell length
L_{sub}	0.02	μm	Distance between junctional SR and surface membrane
R_{cell}	3.8	μm	Cell radius
$V_{i_{part}}$	0.46	-	Cell volume fraction occupied with myoplasm
$V_{jsr_{part}}$	0.0012	-	Cell volume fraction occupied by junctional SR
$V_{nsr_{part}}$	0.0116	-	Cell volume fraction occupied by network SR
V_{cell}	$\pi \cdot R_{cell}^2 \cdot L_{cell}$	μm³	Cell volume
V_{sub}	$2 \cdot \pi \cdot L_{sub} \cdot (R_{cell} – 0.5 \cdot L_{sub}) \cdot L_{cel}$	μm³	Submembrane space
V_i	$V_{i_{part}} \cdot V_{cell} - V_{sub}$	μm³	Myoplasmic volume
V_{jsr}	$V_{jsr_{part}} \cdot V_{cell}$	μm³	Volume of junctional SR
V_{nsr}	$V_{nsr_{part}} \cdot V_{cell}$	μm³	Volume of network SR
Cao	1.8	-	Extracellular Ca^{2+} concentration
Ki	140	-	Intracellular K^+ concentration
Ko	5.4	-	Extracellular K^+ concentration
Nao	140	-	Intracellular Na^+ concentration
Na_i	5.0	-	Extracellular Na^+ concentration
Mgi	2.5	-	Intracellular Mg^{2+} concentration
F	96485	C/mol	Faraday constant
R	8314.472	J/(kmol*K)	Universal gas constant
T	310	K	Absolute temperature for 37°C
RTonF	26.72655	mV	R*T/F
E_{Na}	89.06	mV	Reversal potential for Na^+
E_{mh}	49.83	mV	Reversal potential for fast Na^+
E_K	-87.00	mV	Reversal potential for K^+

E_{Ks}	-49.33	mV	Reversal potential for slow rectifier K^+ channel
E_{Ca}	$0.5 \cdot RTonF - ln\frac{Cao}{Ca_{sub}}$	mV	Reversal potential for Ca^{2+}
g_{fNa}	0.00268	µS	I_f current, Na^+ channel max. conductance
g_{fK}	0.00159	µS	I_f current, K^+ channel max. conductance
P_{CaL}	0.4578	nA/mM	
P_{CaT}	0.04132	nA/mM	
g_{Kr}	0.00424	µS	I_{Kr} current, K^+ channel max. conductance
g_{Ks}	0.00065	µS	I_{Ks} current, K^+ channel max. conductance
$g_{K,ACh}$	0.00345	µS	$I_{K,ACh}$ current, K^+ channel max. conductance
g_{to}	0.0035	µS	I_{to} current, K^+ channel max. conductance
g_{Na}	0.0223	µS	I_{Na} current, Na^+ channel max. conductance
$I_{NaK,max}$	0.08105	nA	max. I_{NaK} current
K_{NaCa}	3.343	nA	Dissociation constant of Ca^{2+}-dependent I_{CaL} inactivation
g_{Kur}	0.0001539	µS	I_{Kur} current, K^+ channel conductance
Km_{fCa}	0.000338	mM	Dissociation constant of Ca^{2+}-dependent I_{CaL} inactivation
Km_{Kp}	1.4	mM	Half-maximal K_o for I_{NaK}
Km_{Nap}	14	mM	Half-maximal Na_i for I_{NaK}
α_{fCa}	0.0075	s^{-1}	Ca^{2+} dissociation rate constant for I_{CaL}
K1ni	395.3	mM	Intracellular Na^+ binding to first site on NaCa
K1no	1628	mM	Extracellular Na^+ binding to first site on NaCa
K2ni	2.289	mM	Intracellular Na^+ binding to second site on NaCa
K2no	561.4	mM	Extracellular Na^+ binding to second site on NaCa
K3ni	26.44	mM	Intracellular Na^+ binding to third site on NaCa
K3no	4.663	mM	Extracellular Na^+ binding to third site on NaCa

Kci	0.0207	mM	Intracellular Ca^{2+} binding to NaCa transporter
Kcni	26.44	mM	Intracellular Na^{+} and Ca^{2+} simultaneous binding to NaCa
Kco	3.663	mM	Extracellular $Ca2^{+}$ binding to NaCa transporter
Qci	0.1369	-	Intracellular Ca^{2+} occlusion reaction of NaCa
Qco	0	-	Extracellular Ca^{2+} occlusion reaction of NaCa
Qn	0.4315	-	Na^{+} occlusion reaction of NaCa
$\tau_{dif_{Ca}}$	$5.469 * 10^{-5}$	s	Time constant of Ca^{2+} diffusion from the subsarcolemmal space to the myoplasm
τ_{tr}	0.04	s	Time constant of Ca^{2+} transfer from the network to junctional SR
K_{up}	286	nM	Half-maximal Cai for Ca^{2+} uptake into the network SR
P_{up}	5	mM/s	Rate constant for Ca^{2+} uptake by SERCA pump into the network SR
$slope_{up}$	50	nM	Slope factor for Ca^{2+} uptake by SERCA pump into the network SR
kiCa	500	$(mM*s)^{-1}$	RyR Ca-dependent inactivation rate
kim	5	s^{-1}	RyR repriming rate
koCa	10000	$(mM^2*s)^{-1}$	RyR Ca-activation rate
kom	660	s^{-1}	RyR deactivation rate
ks	$1.48*10^{8}$	s^{-1}	Ca^{2+} diffusion rate
$EC50_{SR}$	0.45	mM	EC50 for Ca_{jsr}-dependent activation of SR Ca release
HSR	2.5	-	Hill coefficient for Ca_{jsr}-dependent activation of SR calcium release
MaxSR	15	-	Parameter for maximum SR calcium release
MinSR	1	-	Parameter for minimum SR calcium release
CM_{tot}	0.045	mM	Total calmodulin concentration
CQ_{tot}	10	mM	Total calsequestrin concentration
TC_{tot}	0.031	mM	Total concentration of the troponin-Ca^{2+} site
TMC_{tot}	0.062	mM	total concentration of the troponin-Mg^{2+} site

kb_{CM}	542	s^{-1}	Ca^{2+} dissociation constant for calmodulin
kb_{CQ}	445	s^{-1}	Ca^{2+} dissociation constant for calsequestrin
kb_{TC}	446	s^{-1}	Ca^{2+} dissociation constant for the troponin-Ca^{2+} site
kb_{TMC}	7.51	s^{-1}	Ca^{2+} dissociation constant for the troponin-Mg^{2+} site
kb_{TMM}	751	s^{-1}	Mg^{2+} dissociation constant for the troponin-Mg^{2+} site
kf_{CM}	$1.642*10^6$	$(mM*s)^{-1}$	Ca^{2+} association constant for calmodulin
kf_{CQ}	175.4	$(mM*s)^{-1}$	Ca^{2+} association constant for calsequestrin
kf_{TC}	88800	$(mM*s)^{-1}$	Ca^{2+} association constant for the troponin-Ca^{2+} site
kf_{TMC}	227700	$(mM*s)^{-1}$	Ca^{2+} association constant for the troponin-Mg^{2+} site
kf_{TMM}	2277	$(mM*s)^{-1}$	Mg^{2+} association constant for the troponin-Mg^{2+} site
$S_{f,max}$	9.62	mV	Maximum shift of i_f activation curve caused by Ach
$n_{f'}$	0.392	-	Hill coefficient for i_f, affected by [Iso]
$K_{0.5,g_k'}$	19	nM	[Iso] that produces half-maximal increase of g_{Ks}
$f_{K_{max}}$	1.87	-	Relative maximum increase of
$K_{0.5,Ca}$	0.09	nM	[Ach] that produces a half-maximal block of $i_{Ca,L}$
$K_{0.5,Ca'}$	7	nM	[Iso] that produces a half-maximal increase of $i_{Ca,L}$
$K_{0.5,f'}$	13.5	nM	[Iso] that produces a half-maximal shift of i_f activation curve
$S_{Ca',max}$	12.025	mV	Maximum shift of i_{CaL} activation curve caused by Iso
$n_{Ca'}$	0.392	-	Hill coefficient for i_{CaL}, affected by [Iso]
$K_{0.5,Kr'}$	13.5	nM	[Iso] that produces a half-maximal shift of i_{Kr} activation curve
$S_{Kr',max}$	19.24	mV	Maximum shift of i_{Kr} activation curve caused by Iso

n_{Kr}'	0.392	-	Hill coefficient for i_{Kr}, affected by [Iso]

Supplementary Table S8. Model parameter values used for the simulation of the sinoatrial node model. The values used are the same for both the simulation of HTxR and healthy controls.
